# The oleaginous astaxanthin-producing alga *Chromochloris zofingiensis*: potential from production to an emerging model for studying lipid metabolism and carotenogenesis

**DOI:** 10.1186/s13068-021-01969-z

**Published:** 2021-05-15

**Authors:** Yu Zhang, Ying Ye, Fan Bai, Jin Liu

**Affiliations:** Laboratory for Algae Biotechnology and Innovation, College of Engineering, Peking University, Beijing, 100871 China

**Keywords:** Abiotic stress, Astaxanthin, *Chromochloris zofingiensis*, Metabolic engineering, Omics, Triacylglycerol

## Abstract

The algal lipids-based biodiesel, albeit having advantages over plant oils, still remains high in the production cost. Co-production of value-added products with lipids has the potential to add benefits and is thus believed to be a promising strategy to improve the production economics of algal biodiesel. *Chromochloris zofingiensis*, a unicellular green alga, has been considered as a promising feedstock for biodiesel production because of its robust growth and ability of accumulating high levels of triacylglycerol under multiple trophic conditions. This alga is also able to synthesize high-value keto-carotenoids and has been cited as a candidate producer of astaxanthin, the strongest antioxidant found in nature. The concurrent accumulation of triacylglycerol and astaxanthin enables *C. zofingiensis* an ideal cell factory for integrated production of the two compounds and has potential to improve algae-based production economics. Furthermore, with the advent of chromosome-level whole genome sequence and genetic tools, *C. zofingiensis* becomes an emerging model for studying lipid metabolism and carotenogenesis. In this review, we summarize recent progress on the production of triacylglycerol and astaxanthin by *C. zofingiensis*. We also update our understanding in the distinctive molecular mechanisms underlying lipid metabolism and carotenogenesis, with an emphasis on triacylglycerol and astaxanthin biosynthesis and crosstalk between the two pathways. Furthermore, strategies for trait improvements are discussed regarding triacylglycerol and astaxanthin synthesis in *C. zofingiensis*.

## Background

Up to date, the unsustainable fossil fuels have still served as the main global energy sources and their growing consumption leads to increasing emission of carbon dioxide into the atmosphere and thus severe environmental problems that threaten our ecosystem [1]. The utilization of alternative energy sources that are renewable and carbon neutral represents a feasible way toward reducing carbon dioxide emission. Among these energy sources, biofuels are promising alternative to the petroleum-based fuels. Due to the substantial advantages over plant oils for biofuel production, algae-derived oils have received great interest of both academia and industry and been considered as the next-generation biodiesel feedstock with the potential to meet the existing demand for transportation uses [1–4]. During past decades, substantial progress has been achieved in the exploration of algal biodiesel, including algae screening and selection, genetic engineering for trait improvements, and development of technologies for algal cultivation and downstream processes [5, 6]. Nevertheless, to bring down the production cost and realize the commercialization of algal biodiesel, significant challenges remain to be addressed.

In addition to the neutral lipid triacylglycerol (TAG) that is ideal for making biodiesel, algae are able to produce a broad range of value-added compounds, such as high-quality protein, polyunsaturated fatty acids and carotenoids depending on algae species [7–9]. The co-production of these high-value compounds with oils from algae has the potential to add benefits and thus offset the algal biodiesel production cost. Astaxanthin, a secondary keto-carotenoid with the highest antioxidant activity found in nature, is high in price and has been widely explored for food, feed, nutraceutical, and pharmaceutical uses [10–12]. Like TAG, astaxanthin is synthesized and accumulated in certain algae under abiotic stress conditions [13–21]. The characteristic of concurrent accumulation of TAG and astaxanthin makes it feasible to employ algae for integrated production of the two compounds.

*Chromochloris zofingiensis* belongs to green algae and is able to grow robustly to achieve high cell densities under photoautotrophic, heterotrophic and mixotrophic conditions [19, 22–29]. Because of the great capacity in synthesizing TAG (up to 50% of dry weight) under multiple trophic conditions, *C. zofingiensis* is considered as a promising feedstock for biodiesel production [13, 17, 19, 28, 30]. This alga can also synthesize astaxanthin at a volumetric level comparable to that *Haematococcus pluvialis* achieves and has been proposed to serve as an alternative producer of natural astaxanthin [25, 27]. The robust performance in growth and simultaneous accumulation of TAG and astaxanthin in lipid droplets (LDs) enable *C. zofingiensis* an appealing alga for production uses [13, 19, 29, 31, 32]. Recently, the chromosome-level genome sequence of *C. zofingiensis* has been released [33], which, together with the workable genetic tools and random mutagenesis for screening target mutants [34–36], provide unprecedented opportunities to better understand the molecular mechanisms for lipid metabolism and carotenogenesis and the crosstalk between TAG and astaxanthin biosynthetic pathways [14, 18, 37–41]. The review centers around *C. zofingiensis* with an aim to (1) summarize recent progress on TAG and astaxanthin production, (2) update molecular understanding of lipid metabolism, carotenogenesis and the communications between TAG and astaxanthin biosynthesis, and (3) discuss engineering strategies for improving the synthesis of either TAG, astaxanthin or both. Efforts made and underway will turn *C. zofingiensis* into not only a production strain of industrial interest but also an emerging model for fundamental studies on lipid metabolism and carotenogenesis.

## Taxonomy, morphology and ultrastructure of *C. zofingiensis*

**Fig. 1:**
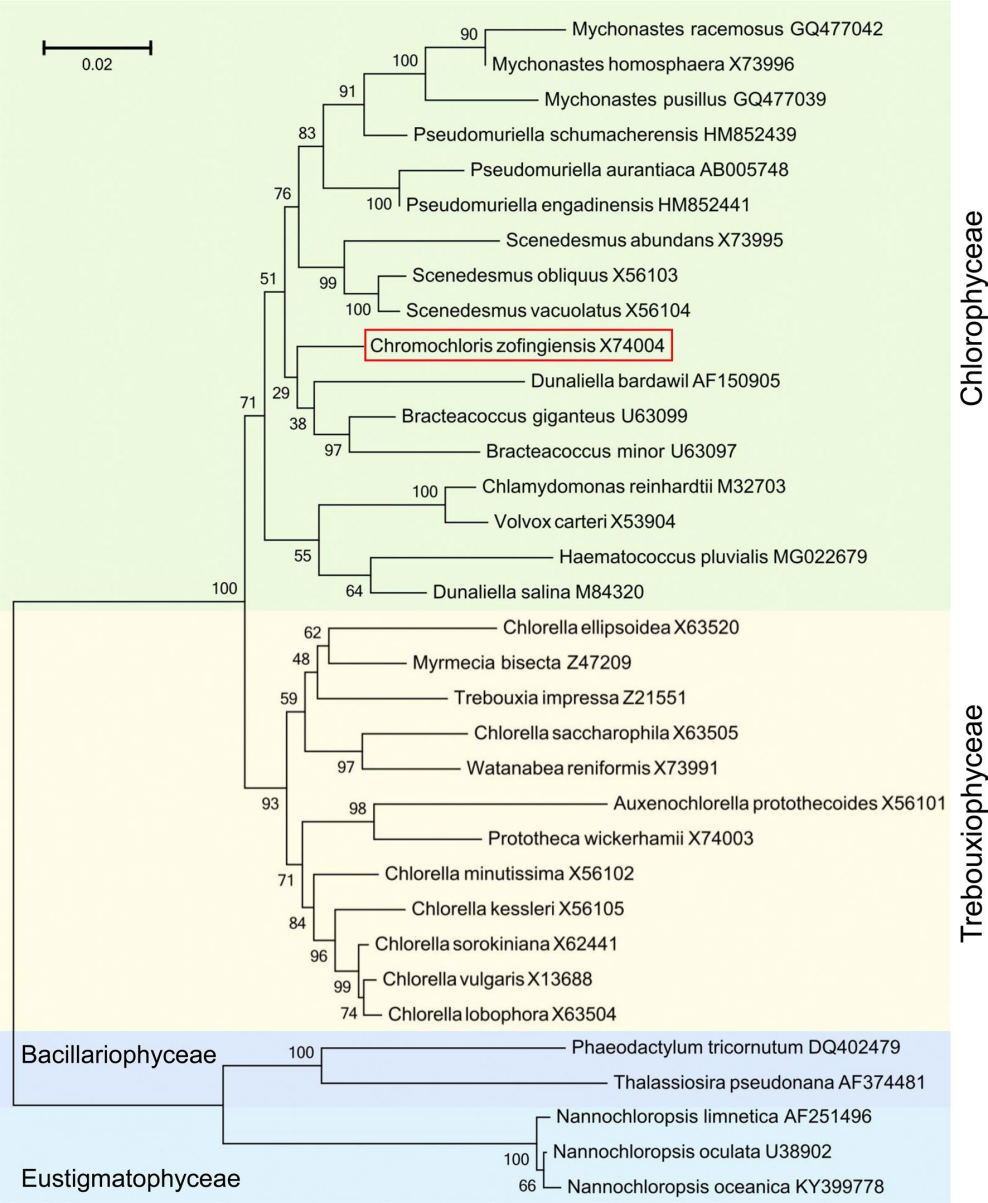
Phylogenetic tree based on the 18S rRNA gene sequences showing relationships of *C. zofingiensis* to other algae. Alignment of sequences was conducted using ClustalX 2.1. The tree was generated in the MEGA6.0 software using the maximum-likelihood method, with the bootstrap value (obtained from 1000 replicates) is shown on each node. The scale bar 0.02 represents 2% divergence, calculated as the estimated number of replacement. The GenBank IDs of 18S rRNA gene sequences are right behind the name of algal species

*C. zofingiensis* is a freshwater green alga and has a complicated taxonomic history. It was isolated in 1934 by Dönz and was originally assigned to the Genus *Chlorella* [42]. Based on detailed observations of morphology and life cycle, Hindák claimed that *C. zofingiensis* was more similar to *Muriella aurantiaca* than to the *Chlorella* type species *Chlorella vulgaris* and thus was recommended to be assigned under the Genus *Muriella* [43]. Afterwards, the taxonomy of this alga was reconsidered and placed under the Genus *Mychonastes* based on scanning and transmission electron microscope observations [44]. Nevertheless, the phylogenetic analyses using genetic sequences, such as the nuclear small subunit (18S) rRNA and/or the nuclear ribosomal internal transcribed spacer 2 (ITS2), suggested that *C. zofingiensis* is distinct from either *Chlorella* [45], *Muriella* [46] or *Mychonastes* [47]. To resolve the uncertain phylogenetic position of *C. zofingiensis*, Fučíková and his co-worker adopted both morphologic observations and genetic sequences of 18S rRNA, ITS2, the large subunit of ribulose 1,5-bisphosphate carboxylase/oxygenase (rbcL) and the plastid-encoded elongation factor TU (tufA), and put *C. zofingiensis* together with *Bracteacoccus cinnabarinus* and *Bracteacoccus minutus* under the genus *Chromochloris* [48]. A phylogenetic tree based on the 18S rRNA sequences is shown in Fig. 1; although in the same Class Chlorophyceae, *C. zofingiensis* is somewhat distant from the other astaxanthin-producing alga *H. pluvialis*.

**Fig. 2:**
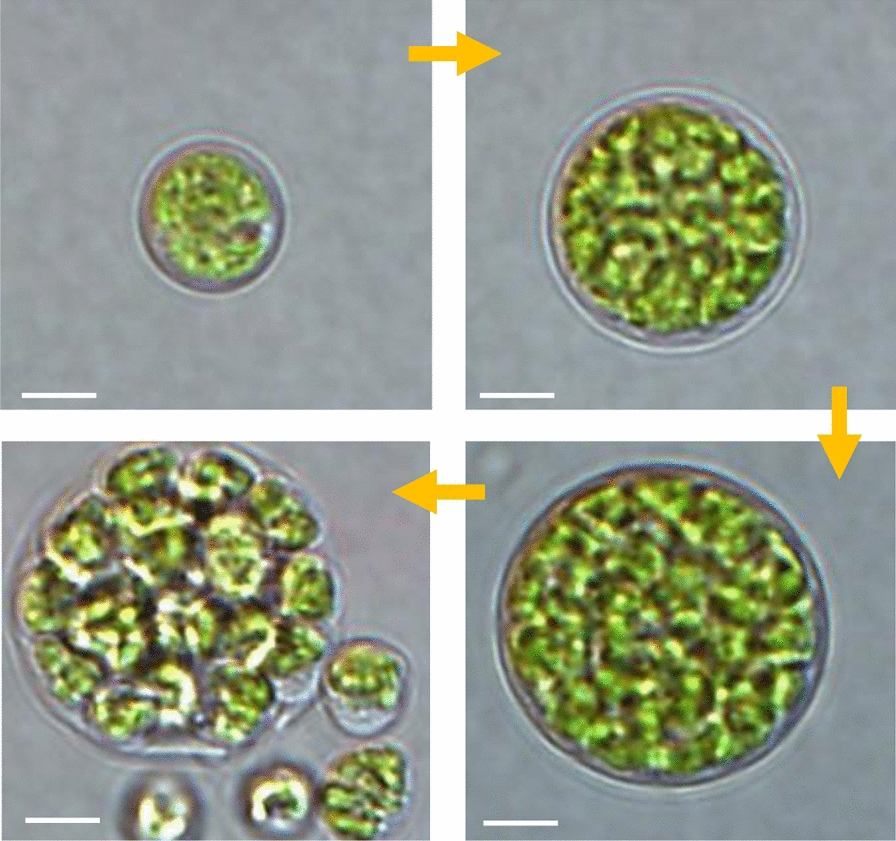
Light microscopic observation of *C. zofingiensis* cells under different growth stages. Bar, 2 μm

*C. zofingiensis* cells are in unicellular and spherical form without flagellum and the cell size in diameter normally ranges from 2 to 15 μm depending on the growth conditions and stages [49]. *C. zofingiensis* is a haploid alga and can reproduce itself via asexual multiple fission. Sexual reproduction has never been observed in this alga. The life cycle of *C. zofingiensis* is simple and generally involves three phases of growth, ripening, and division (Fig. 2). The multiple fission cell cycle of *C. zofingiensis*, resembling *Scenedesmus* and *Desmodesmus*, is in the consecutive pattern, under which DNA replication and nuclear division are executed multiple times prior to cell division [50]. Therefore, polynuclear cells are observed for *C. zofingiensis* and the number of nucleus within a cell is determined by the number of DNA replication and nuclear division events before cell division. When the parental cell wall ruptures, autospores (up to 32) are released spontaneously and enter into the next multiple fission cell cycle [50]. By contrast, *C. reinhardtii* has a clustered pattern of multiple fission cell cycle, under which cell division occurs right after nuclear division; therefore, *C. reinhardtii* generally does not include polynuclear stages [51].

**Fig. 3:**
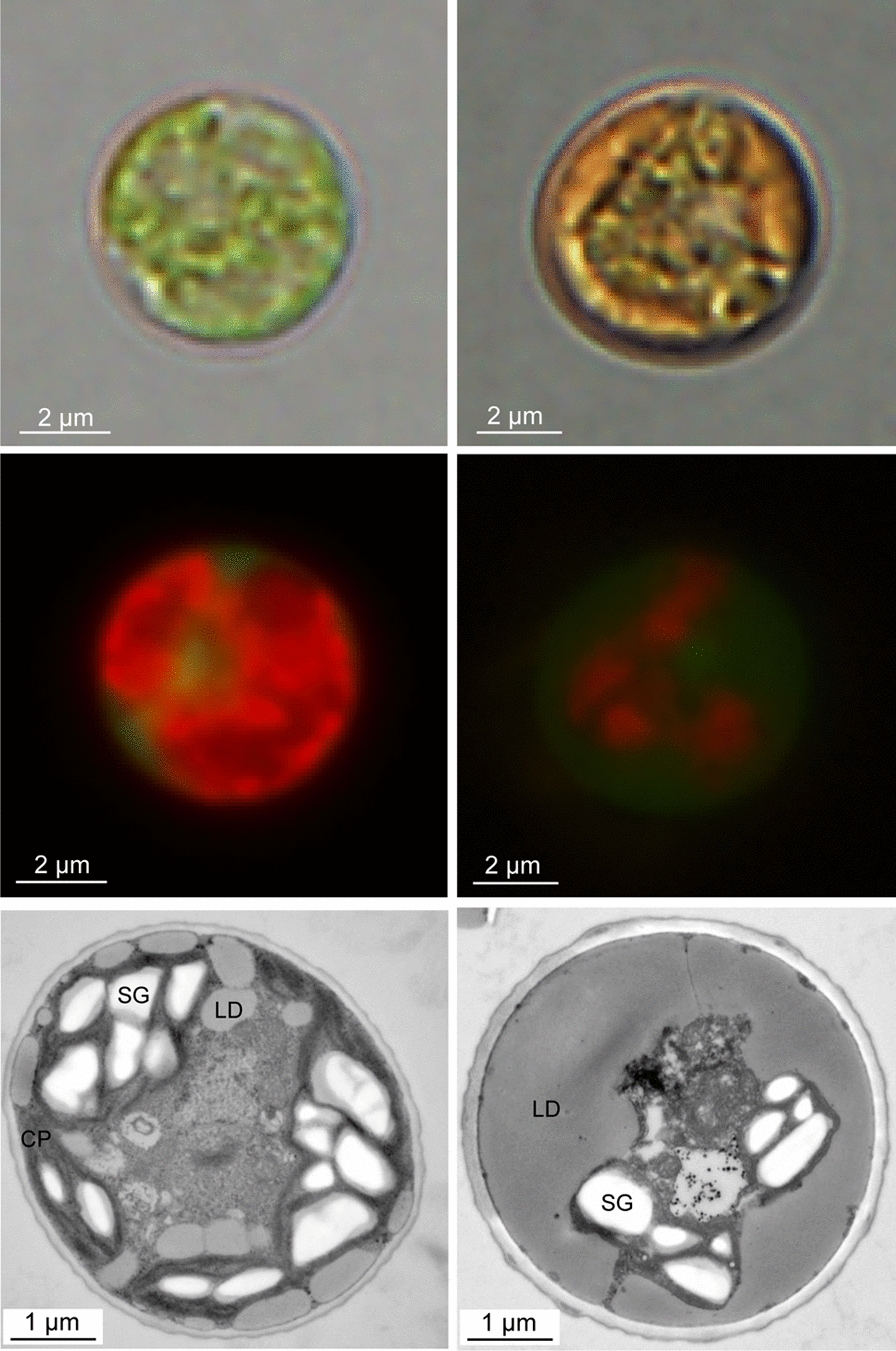
Microscopic observation of *C. zofingiensis* cells under favorable (left) and stress (right) growth conditions. Up, light microscopy; middle, fluorescent microscopy (red indicates chlorophyll autofluorescence and green indicates neutral lipids stained with BODIPY); bottom, transmission electron microscopy. CP, chloroplast; LD, lipid droplet; SG, starch granule

*C. zofingiensis* possesses a rigid cell wall, which is mainly composed of glucose and mannose and tends to get thicker under stress conditions [52–55] (Fig. 3). *C. zofingiensis* cells appear green under favorable growth conditions and turn orange under stress conditions (Fig. 3), due to the induction of secondary carotenoids including astaxanthin [13, 19, 22, 54, 55]. Observations based on transmission electron microscopy suggest that *C. zofingiensis* has a cup-shaped chloroplast sitting peripherally in the cytoplasm, which contains no pyrenoid but scattered starch granules; small LDs are also present and closely associated with the chloroplast (Fig. 3). Stress conditions severely impact the ultrastructure of *C. zofingiensis* cells, leading to the shrunken chloroplast, decreased starch granules and expanded LDs that embrace the chloroplast (Fig. 3). The close proximity of the keto-carotenoids-containing LDs to the cell wall indicates that secondary carotenoids may serve as substrates for synthesizing sporopollenin in cell walls, as is the case in other astaxanthin-producing algae [55, 56].

## *C. zofingiensis* as a promising producer of lipids and carotenoids

### Growth physiology and trophic modes

*C. zofingiensis* requires certain nutrients to support its growth, including carbon, nitrogen, phosphorus, and inorganic salts. Carbon is the most prominent element and accounts for approximately 50% of the algal biomass. *C. zofingiensis* is able to utilize both inorganic and organic carbon sources. Carbon dioxide (CO_2_) is the primary inorganic carbon source for algal growth and it has been reported that some algae can tolerate high CO_2_ level of ~ 40% [1]. There is no report about the tolerance ability of *C. zofingiensis* to CO_2_ level. In general, a concentration of 0.5–2% CO_2_ (mixed with air by volume) is supplied to sustain photoautotrophic growth of *C. zofingiensis*, giving rise to a dry biomass density of ~ 13.5 g L^−1^ in batch cultures [13, 17–20, 22, 32, 55, 57, 58]. Light is indispensable for photoautotrophic growth of algae. *C. zofingiensis* has the ability to maintain its growth under high light intensities (~ 1500 μE m^−2^ s^−1^), suggesting the feasibility of growing this alga outdoors with strong sunlight for mass production [58]. This excellent adaption to high light may be due to the strong non-photochemical quenching ability *C. zofingiensis* possesses [59]. Within the saturation light range, *C. zofingiensis* growth is dependent on the light intensity: the higher the light intensity, the greater the biomass achieved [27, 57, 58, 60].

Nitrogen, the important element of protein, is crucial for algal growth. Nitrate, urea and ammonia represent the most commonly used nitrogen sources. *C. zofingiensis* can utilize both nitrate and urea well for growth, but grows poorly with ammonia [61, 62]. The poor growth is probably due to the acidification of the culture medium resulting from the consumption of ammonia, which has been reported for other algae [28, 63–65]. Nitrogen concentration in the culture medium plays an important role in affecting algal growth. It has been reported that nitrogen limitation/starvation impairs the growth of *C. zofingiensis* severely, accompanied by the enlargement of cell size [13, 17, 21, 22, 41]. Phosphorus is also an important element required for sustaining algal growth. Nevertheless, phosphorus is less prominent than nitrogen on algal growth and phosphorus limitation/starvation causes only a moderate growth impairment for *C. zofingiensis* [8, 17]. It is worth noticing that the micronutrient sulfur has a greater effect than phosphorus on *C. zofingiensis* growth, as suggested by the more severely impaired growth under sulfur starvation compared to under phosphorus starvation [17]. As a freshwater alga, *C. zofingiensis* is able to tolerate moderate salt levels (~ 0.25 M NaCl), yet at the expense of growth [18, 32].

*C. zofingiensis* can utilize various organic carbon sources, such as sugars, acetate and glycerol for heterotrophic growth, of which glucose is the most widely used one [23, 30, 31]. By contrast, *H. pluvialis* cannot utilize glucose but acetate for efficient heterotrophic growth [66], probably due to the lack of glucose transporter that is responsible for importing glucose from the medium [67]. In batch cultures, *C. zofingiensis* growth is affected by glucose concentration in the medium, and the final algal biomass yield correlates positively with the initial glucose concentration within the range of 0–30 g L^−1^ [23, 27]. Nevertheless, high glucose concentration has adverse effect on algal growth. To address this, fed-batch cultivation can be employed, in which glucose is fed into the culture medium time by time to maintain its concentration below a certain level, e.g., 20 g L^−1^, achieving an ultrahigh algal biomass density of ~ 100 g L^−1^ [25–27, 30, 68]. The ultrahigh fermented *C. zofingiensis*, with or without dilution, can be used as seed cultures for photoautotrophic growth and carotenogenesis [27, 68]. Furthermore, *C. zofingiensis* grows well under mixotrophic conditions in the presence of light illumination, where both organic (glucose or acetate) and inorganic carbon sources are provided [21, 24, 29, 62, 69, 70]. It has been proposed that the mixotrophic cultivation has synergistic effect on growth and biomass production of *C. zofingiensis* [69].

### Lipid production

Lipids can be roughly clarified as polar lipids, e.g., phospholipids and glycolipids that are the main constitutes of various membranes, and neutral lipids, e.g., TAG that is the most energy-dense storage lipid. Under favorable growth conditions, algae contain predominantly polar membrane lipids with only a basal level of TAG; upon stress conditions, algae tend to slow down growth and accumulate TAG in bulk as the carbon and energy reservoir [3]. These stress conditions include but are not restricted to limitation/starvation of nutrients (e.g., nitrogen, phosphorus, sulfur, iron and zinc), high light, salinity, and abnormal temperature [13, 17, 18, 71–78].

The use of *C. zofingiensis* for lipid production has been widely assessed in the past decade [13, 17–20, 28, 30, 31, 35, 60, 62, 70, 79–82]. Although lipid accumulation in *C. zofingiensis* has long been observed via transmission electron microscopy [55], lipid quantification of this alga was not performed until 2010 by Liu and his co-workers [30]. This pioneering work examined the effect of various sugars (lactose, galactose, sucrose, fructose, mannose and glucose) on lipid production by heterotrophic *C. zofingiensis* and found that glucose is superior to other sugars for lipid content and yield. The lipid content in *C. zofingiensis* reached ~ 52% of dry weight, of which TAG accounted for 72%. Fed-batch cultivation was also conducted for *C. zofingiensis*, giving rise to 20.7 g L^−1^ and 1.38 g L^−1^ d^−1^ for lipid yield and productivity, respectively. Nevertheless, the need of glucose makes lipid production from *C. zofingiensis* less economically viable, particularly for making the low-value commodity biodiesel, driving the exploration of such alternative and cheap carbon sources from cellulosic materials and industrial waste sugars [83–85]. Liu et al. [31] assessed the use of cane molasses, a waste of the sugar industry, for heterotrophic lipid production by *C. zofingiensis.* The results suggested that cane molasses, after proper pretreatment, could be used as a substitute of glucose to support *C. zofingiensis* for achieving high biomass and lipid productivities. It is worth noting that the sugar-to-lipid conversion ratio is generally below 25% for heterotrophic *C. zofingiensis* cultures [30, 31, 79], raising the challenge regarding how to improve the sugar-based lipid yield.

Concerning photoautotrophic lipid production, Mulders et al. [19] assessed *C. zofingiensis* cultures under nitrogen deprivation (ND) conditions, in which TAG content and yield reached 0.34 g mg^−1^ dry weight and 2.9 g L^−1^, respectively. Later, Liu et al. [13] compared lipid production performance by photoautotrophic *C. zofingiensis* under various conditions of ND, high light (HL) and the combination of ND and HL (ND + HL). ND + HL enabled *C. zofingiensis* to produce the highest levels of total lipids and TAG, followed by ND and HL. Nevertheless, due to the compromised biomass production, TAG productivities achieved under ND and ND + HL conditions were lower than that under HL conditions. To promote TAG productivity, the authors employed a nitrogen limitation strategy coupled with a semi-continuous culture system. The effect of other nutrients, such as phosphorus and sulfur, was also evaluated for *C. zofingiensis*: similar to ND, sulfur deprivation (SD) induced TAG accumulation yet less prominent; by contrast, phosphorus deprivation (PD) showed little impact on TAG synthesis [17]. Interestingly, other algae, such as *Nannochloropsis* and *Phaeodactylum,* are vulnerable to PD for TAG induction [74, 86, 87], highlighting the evolutionary divergence of these algae in sensing and responding to phosphorus changes. *C. zofingiensis* is able to grow in the presence of moderate salinity levels [18, 22, 88]. As shown in other green algae [89–92], *C. zofingiensis* was reported to synthesize and accumulate TAG upon salinity stress (SS) [18], pointing to the potential of using this alga for lipid production under saline environment, thus reducing freshwater footprint. Furthermore, the combination of HL and SS (HL + SS) was shown to induce more TAG in *C. zofingiensis* and give rise to higher TAG yield and productivity than HL and SS alone did [32]. In addition, it has been recently reported that lipid accumulation in *C. zofingiensis* could be stimulated by certain phytohormones, resulting in enhanced lipid yield and productivity [29]. A summary of lipid production by *C. zofingiensis* under various conditions is listed in Table 1. There are a number of reviews about lipid production by microalgae during the past decades; the lipid content and lipid productivity, depending on microalgal species/strains and culture conditions, normally range from 20 to 60% of dry weight and 30 to 600 mg L^−1^ d^−1^, respectively [3, 93–95]. It may be not appropriate to conclude by direct comparison of lipid content and productivity between *C. zofingiensis* and other algae, as the culture conditions are different. Nevertheless, the TAG content (~ 48% of dry weight), yield (~ 20.4 g L^−1^) and productivity (~ 1.4 g L^−1^ day^−1^) achieved for *C. zofingiensis* are overall comparable to or even higher than those from other commonly studied and potential lipid production algae, such as *Chlorella*, *Scenedesmus*, *Nannochloropsis*, etc. [28, 94, 96–99].

**Table 1:** Summary of TAG and astaxanthin production by *C. zofingiensis*

References	Culture conditions	Biomass		TAG or TFA			Astaxanthin		
		Concentration (g L^−1^)	Productivity (g L^−1^ day^−1^)	Content (g g^−1^DW)	Yield (g L^−1^)	Productivity (g L^−1^ day^−1^)	Content (mg g^−1^DW)	Yield (mg L^−1^)	Productivity (mg L^−1^ day^−1^)
[88]	P, Batch	–	–	–	–	–	6.8	–	0.8
[22]	P, Batch	7.0	0.7	–	–	–	3.7	25.0	1.3
[24]	M, Batch	9.5	–	–	–	–	1.3	12.5	–
[26]	H, FB	53.0	3.3	–	–	–	0.7	32.4	2.0
[30]	H, FB	43.1	2.9	0.48^a^	20.7	1.4	–	–	–
[31]	H, Batch	12.9	1.6	0.33	4.2	0.5	1.2	13.6	1.7
[25]	H, FB	45.6	4.7	–	–	–	1.2	56.1	5.6
[77]	M, Batch	11.9	0.6	0.42^a^	5.0	0.3	2.2	25.8	1.3
[19]	P, Batch	8.2	0.6	0.34	2.8	0.3	2.4	19.6	1.4
[13]	P, Batch P, SC	7.5 –	1.4 1.0	0.39 0.29	1.5 –	0.2 0.3	4.9 3.2	12.8 –	2.0 3.3
[21]	M, Batch	6.0	0.5	–	–	–	6.5	38.9	3.2
[68]	M, TS	98.4	7.0	–	–	–	0.8	73.3	5.2
[17]	P, Batch	1.8	0.2	0.27	0.4	0.1	3.9	4.5	0.6
[27]	H, FB H-P, TS	71.1 73.7	5.8 4.8	– –	– –	– –	0.7 2.7	47.3 194.5	4.0 9.9
[29]	M, Batch	8.3	0.7	0.65^a^	5.4	0. 5	13.1	89.9	7.5
[18]	P, Batch	3.8	1.0	0.19	0.5	0.1	3.0	6.8	1.7
[32]	P, Batch	7.2	1.1	0.42^a^	3.0	0.5	6.0	41.8	7.0
[70]	M, FB	7.8	1.1	0.42^a^	–	0.46	–	–	2.0

^a^ TFA; P, photoautotrophic culture; M, mixotrophic culture; H, heterotrophic culture; SC, semi-continuous culture; FB, fed-batch culture; TS, two-stage cultivation

The fatty acid composition of lipids is also important, as it determines key properties of biodiesel, such as cetane number, heat of combustion, oxidative stability, cloud point, lubricity [100]. Similar to plant oils, *C. zofingiensis* lipids consist predominantly of fatty acids in the length of 16–18 carbons [30]. The relative abundance of fatty acids in *C. zofingiensis* varies largely depending on the culture conditions [13, 17, 18, 28, 29, 31, 62, 79]. In general, saturated fatty acids provide oxidative stability, while unsaturated fatty acids benefit low-temperature stability. It is believed that oleic acid (C18:1^∆9^) can serve as a balance between oxidative stability and low-temperature performance, and its high abundance is beneficial to biodiesel quality [100, 101]. In *C. zofingiensis*, C18:1^∆9^ abundance correlates positively with TAG content and its relative abundance in TAG can reach ~ 60% [13, 17, 18, 30, 31], pointing to the potential of using lipids from this alga for making high-quality biodiesel.

### Carotenoid production

Carotenoids, the abundant natural pigments, are widely distributed in photosynthetic organisms, some non-photosynthetic bacteria and fungi [102]. The common carotenoids found in vascular plants, e.g., β-carotene, zeaxanthin, neoxanthin, antheraxanthin, violaxanthin, α-carotene and lutein, are also present in green algae. These primary carotenoids serve as important components of photosynthetic apparatus and are critical for photoautotrophic growth. Aside from primary carotenoids, some green algae synthesize keto-carotenoids (also called secondary carotenoids), such as echinenone, canthaxanthin, adonirubin, adonixanthin, astaxanthin and keto-lutein [8, 54, 55, 88, 103–107]. Distinct from primary carotenoids, secondary carotenoids are synthesized in large quantities by certain algae only under specific stress conditions and generally reside in the extrachloroplastic organelle lipid body (LD) [40, 55, 108, 109]. Among the secondary carotenoids, astaxanthin possesses the strongest antioxidant activity with broad applications and has long been receiving interests of both academia and industry [10, 56, 110, 111]. So far, *H. pluvialis* is the only alga used for commercial production of astaxanthin. Nevertheless, slow growth rate, low biomass production and ease of contamination by other fast-growing organisms restrict the yield of astaxanthin from *H. pluvialis*, driving the exploration of alternative algal producers, e.g., *C. zofingiensis* [8].

In addition to astaxanthin, *C. zofingiensis* synthesizes a series of other keto-carotenoids including echinenone, canthaxanthin, adonixanthin and keto-lutein [8, 107]. Astaxanthin production from photoautotrophic *C. zofingiensis* cultures has long been studied [54, 55, 104, 112]. In these early works, the only recorded secondary carotenoids were astaxanthin (~ 70%) and canthaxanthin (~ 30%). Later, Del Campo et al. [22] evaluated the effect of different environmental and nutritional factors (i.e., temperature, light intensity, salinity level and nitrate concentration) on astaxanthin production by *C. zofingiensis* and achieved a maximum astaxanthin yield of 25 mg L^−1^ and productivity of 1.3 mg L^−1^ day^−1^. In the study conducted by Mulders et al. [19], the ND-induced *C. zofingiensis* accumulated astaxanthin, canthaxanthin, and keto-lutein as the main secondary carotenoids; the astaxanthin content, yield and productivity acheieved were 2.4 mg g^−1^ dry weight, 20 mg L^−1^ and 1.4 mg L^−1^ day^−1^, respectively. Comparatively, among the three nutrient stress conditions of ND, PD and SD, ND enabled *C. zofingiensis* to synthesize the highest level of astaxathin (3.9 mg g^−1^ dry weight), followed by SD and PD [17]. The effect of stress conditions alone or in combination on astaxanthin production by *C. zofingiensis* has also been comparatively examined [13, 32]. Apparently, ND + HL was demonstrated to be more efficient than ND or HL alone for astaxanthin induction in *C. zofingiensis*, giving rise to an astaxanthin content of 4.9 mg g^−1^ dry weight in a 6-day batch culture [13]. Nevertheless, the astaxanthin productivity was compromised by the impaired growth under ND + HL and thus just comparable to that under HL (2.0 versus 1.8 mg L^−1^ day^−1^) [13]. Similarly, HL + SS was shown to surpass HL or SS alone in inducing astaxanthin synthesis and allowed *C. zofingiensis* to accumulate astaxanthin at a level of ~ 6.0 mg g^−1^ dry weight [32]. Unlike ND + HL, HL + SS was also superior to HL or SS alone and gave rise to the greatest astaxanthin yield (41.8 mg L^−1^) and productivity (7.0 mg L^−1^ day^−1^) [32]. Astaxanthin content in *C. zofingiensis* could be further promoted to 6.8 mg g^−1^ dry weight under the combination of three stress conditions, i.e., HL, ND and SS, yet astaxanthin productivity was low (0.8 mg L^−1^ day^−1^) because of the severely impaired growth [88].

Heterotrophic production of astaxanthin from *C. zofingiensis* has also been intensively studied, using sugars particularly glucose as the sole carbon and energy source [23, 25–27, 31, 68, 113, 114]. Concerning heterotrophic *C. zofingiensis* cultures, sugar concentration or carbon/nitrogen (C/N) ratio in the culture medium correlates with astaxanthin content in the alga, e.g., as sugar concentration increased from 5 g L^−1^ to 50 g L^−1^, astaxanthin content rose from 0.44 to 1.01 mg g^−1^ dry weight [23]. Reactive oxygen species and reactive nitrogen species were shown to promote astaxanthin accumulation in heterotrophic *C. zofingiensis* cells [113, 114]. Of six sugars tested, glucose and mannose were more effective than other four for inducing astaxanthin accumulation in *C. zofingiensis* batch cultures; using the glucose-based fed-batch cultivation (15-day period), biomass concentration and astaxnathin yield increased from 10.3 g L^−1^ and 10.5 mg L^−1^ to 51.8 g L^−1^ and 32.4 mg L^−1^, respectively [26]. Later, the fed-batch cultivation of *C. zofingiensis* using pretreated molasses was performed, in which astaxanthin yield and productivity after 10 days of cultivation reached 45.6 mg L^−1^ and 5.35 mg L^−1^ day^−1^, respectively [25]. In another fed-batch fermentation study (14-day period), the authors reported even higher biomass concentration and astaxanthin yield, which were 98.4 g L^−1^ and 73.3 mg L^−1^, respectively [68]. Albeit with ultrahigh biomass concentration, these heterotrophic *C. zofingiensis* cultures contained astaxanthin below 1.0 mg g^−1^ dry weight [25, 26, 68], much less than that achieved in photoautotrohphic cultures [13, 17, 19, 32, 88]. Likely, light is a key inducer for enhancing astaxanthin accumulation in *C. zofingiensis*. In this context, Sun et al. [27] developed a novel heterotrophy − photoinduction culture strategy for *C. zofingiensis*: the alga was first cultured in a heterotrophic fed-batch mode for achieving ultrahigh biomass density, followed by transfer of the heterotrophic cultures without dilution to light for photoinduction of astaxanthin. This strategy enabled *C. zofingiensis* to produce 2.6 mg g^−1^ astaxanthin and so far the highest astaxanthin yield and productivity, i.e., 194.5 mg L^−1^ and 9.9 mg L^−1^ day^−1^.

There have been several reports about using mixotrophic *C. zofingiensis* cultures for astaxanthin production [21, 24, 29, 77]. In the study conducted by Chen et al. [21], *C. zofingiensis* was cultured with a high C/N ratio in the presence of HL, and astaxanthin content, yield and productivity achieved were 6.5 mg g^−1^, 38.9 mg L^−1^ and 3.24 mg L^−1^ day^−1^, respectively. It has been suggested that phytohormones can be employed in combination with stress conditions to enhance astaxanthin accumulation in *H. pluvialis* [115]. Similarly, certain phytohormones were shown to promote astaxanthin production by *C. zofingiensis* under mixotrophic growth conditions, with astaxanthin content, yield and productivity being 13.1 mg g^−1^, 89.9 mg L^−1^ and 7.49 mg L^−1^ day^−1^, respectively [29]. The detailed summary of astaxanthin production by *C. zofingiensis* under various conditions is listed in Table 1. Albeit the highest astaxanthin content obtained for *C. zofingiensis* (13.1 mg g^−1^ dry weight) is still much lower than that for *H. pluvialis* (> 40 mg g^−1^ dry weight), the astaxanthin yield (~ 194.5 mg L^−1^) and productivity (~ 9.9 mg L^−1^ day^−1^) for *C. zofingiensis* are comparable to and in some cases higher than that of *H. pluvialis* [116–121].

Natural astaxanthin has free and esterified forms. Astaxanthin-producing algae, with a couple of exceptions that produce only free form [105, 122], accumulate both forms and the relative proportions depend on the algae species and culture conditions [8, 56, 104]. It has been suggested that esterified astaxanthin is more stable and has stronger antioxidant ability than free astaxanthin [123, 124]. *C. zofingiensis* accumulates esterified astaxanthin as the major proportion, which can reach ~ 92% of total astaxanthin and ~ 70% of total secondary carotenoids under induction conditions [13, 14, 17, 32, 55, 104, 107].

### Simultaneous production of TAG and astaxanthin

It is believed that integrated production of TAG with high-value products from algae has the potential to improve algal biodiesel production economics [7]. The implementation of this concept, from a biorefinery point of view, requires simultaneous accumulation of TAG and high-value products in algae. The high-value carotenoid astaxanthin, similar to TAG, belongs to secondary metabolites and is stored in LDs in algae [40, 109]. In *C. zofingiensis* both TAG and astaxanthin are induced to synthesize and accumulate under certain above-mentioned conditions, such as ND, SD, HL, SS, ND + HL, HL + SS, high sugar concentration [13, 14, 17–19, 29, 31, 32, 62]. Specifically, when plotting TAG contents with astaxanthin contents from different time points of each condition, a strong linear relationship was observed with the *R*^2^ being over 0.975 [13, 14]. This reflects the coordinated and simultaneous accumulation of TAG and astaxanthin in *C. zofingiensis* and guarantees the feasibility of using this alga for integrated production of the two compounds. In this context, *C. zofingiensis* has the potential to serve as a leading algal producer of lipids for biodiesel and an alternative promising source of natural astaxanthin.

### Extraction of TAG and astaxanthin

Considering that both TAG and astaxanthin are stored in LDs of *C. zofingiensis* [40], co-extraction of these two compounds from the alga is possible. Nevertheless, *C. zofingiensis* possesses rigid cell wall particularly under stress conditions [8] and thus cell disruption is required to facilitate extraction of TAG and astaxanthin from the alga and downstream processes. Many mechanic and non-mechanic disruption methods have been developed and applied to rupture cell walls of various microalgae; the former include bead beating [125], grinding [126], ultrasonication [127], high-pressure homogenization [128] and expeller pressing [129], and the latter include repeated freeze–thaw [130], osmotic shock [131], microwave radiation [132] and enzymatic digestion [133]. These methods should also work for cell wall disruption of *C. zofingiensis*, though modifications may be needed due to differences in cell wall composition and rigidity between *C. zofingiensis* and other algae [134].

Organic solvents can be applied to ruptured algal cells for easy extraction of lipids and pigments. The frequently used organic system for *C. zofingiensis* is a mixture of chloroform and methanol (2:1, v/v), which has been demonstrated to extract both TAG and astaxanthin efficiently [13, 14, 17]. Nevertheless, this polar organic mixture extracts not only TAG and astaxanthin but also polar lipids. Low-polarity organic solvents, such as hexane/isopropanol, have been used for highly selective extraction of TAG from microalgae [135, 136]. This should work for *C. zofingiensis* to selectively extract TAG as well as astaxanthin. As the use of organic solvents brings environmental and safety issues, alternative green solvents, such as supercritical fluids (e.g., CO_2_) and ionic liquids, have emerged as the extraction media for lipids from microalgal biomass [137–140]. Whether these methods can be applied to *C. zofingiensis* for efficient TAG and astaxanthin extraction needs to be experimentally evaluated.

## Lipid metabolism in *C. zofingiensis*

Although the past decade has witnessed substantial progress in lipid production by *C. zofingiensis*, the content and yield need to be improved for more viable biodiesel uses, which rely on genetic modifications of the alga guided by deep understanding of lipid metabolism. The availability of *C. zofingiensis* genome sequence [33] and knowledge from *C. reinhardtii*, a close relative to *C. zofingiensis* with detailed study on acyl-lipid metabolism [141–143], accelerate research and understanding on lipogenesis for TAG biosynthesis in *C. zofingiensis*.

### Profiles of fatty acids and glycerolipid classes

**Fig. 4:**
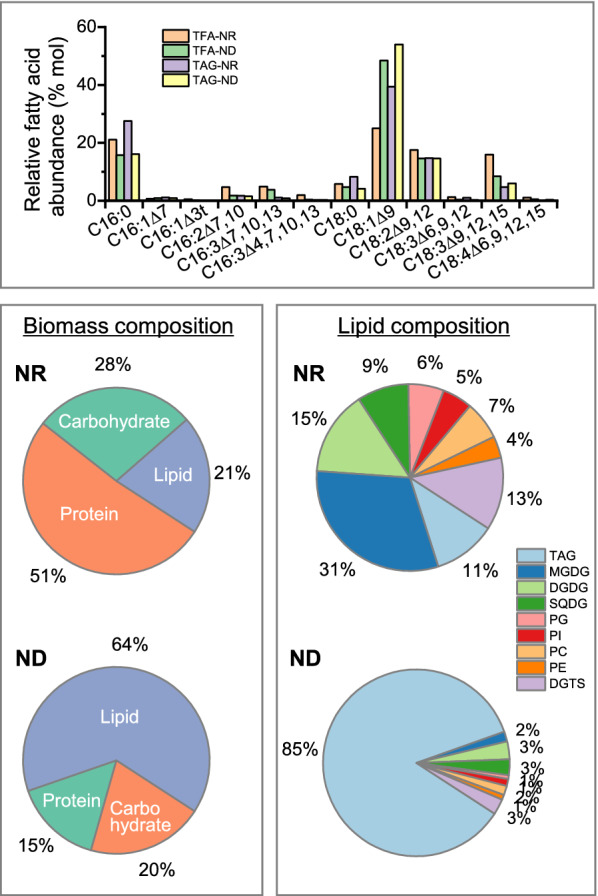
Profiles of fatty acids and glycerolipids in *C. zofingiensis* under nitrogen replete (NR) and nitrogen deprivation (ND) conditions. DGDG, digalactosyl diacylglycerol; DGTS, diacylglycerol-*N*,*N*,*N*-trimethylhomoserine; MGDG, monogalactosyl diacylglycerol; SQDG, sulfoquinovosyl diacylglycerol; PE, phosphatidylethanolamine; PG, phosphatidylglycerol; PI, phosphatidylinositol; TAG, triacylglycerol; TFA, total fatty acids

The fatty acid profile of *C. zofingiensis* has been determined and reported by numerous studies in the past decade [13, 17, 18, 28–32, 37, 62, 79]. In general, the fatty acids are composed of C16:0, C16:1^∆7^, C16:1^∆3t^, C16:2^∆7,10^, C16:3^∆7,10,13^, C16:3^∆4,7,10,13^_,_ C18:0, C18:1^∆9^, C18:2^∆9,12^, C18:3^∆6,9,12^, C18:3^∆9,12,15^, and C18:4^∆6,9,12,15^ (Fig. 4). This differs from the fatty acid composition of *C. reinhardtii* in which C18:3^∆6,9,12^ and C18:4^∆6,9,12,15^ are replaced by C18:3^∆5,9,12^ and C18:4^∆5,9,12,15^, respectively [141]. The relative abundance of fatty acids in *C. zofingiensis* varies greatly depending on culture conditions, for example, the major monounsaturated fatty acid C18:1^∆9^ has a considerably higher percentage under ND + HL than under favorable growth conditions, with a lower percentage of polyunsaturated fatty acids [13].

In addition to the polar glycerolipids present in *C. reinhardtii*, e.g., monogalactosyl diacylglycerol (MGDG), digalactosyl diacylglycerol (DGDG), sulfoquinovosyl diacylglycerol (SQDG), phosphatidylglycerol (PG), phosphatidylinositol (PI), phosphatidylethanolamine (PE) and diacylglycerol-*N*,*N*,*N*-trimethylhomoserine (DGTS), *C. zofingiensis* contains phosphatidylcholine (PC) as well [18, 37, 38]. As indicated in Fig. 4 based on the data from Liu et al. [37], under nitrogen-replete favorable growth conditions, the lipid fraction accounts for only a small proportion of cell mass, of which membrane lipids particularly the glycolipids MGDG and DGDG are the major lipid classes. By contrast, under such stress condition as ND, the lipid fraction dominates the proportion of cell mass, contributed by the huge increase of TAG. Polar lipids, on the other hand, decrease severely in their proportion.

### Fatty acid biosynthesis, desaturation and degradation

Green algae, similar to vascular plants, perform de novo fatty acid synthesis in the chloroplast, using acetyl-CoA as the precursor and building block [141]. Multiple routes are proposed for producing acetyl-CoA: from pyruvate mediated by pyruvate dehydrogenase complex (PDHC), from pyruvate via PDHC bypass, from citrate through the ATP-citrate lyase (ACL) reaction, and from acetylcarnitine via carnitine acetyltransferase reaction [144]. *C. zofingiensis* genome harbors genes encoding enzymes involved in the first three routes [37]. Taking into account the predicted subcellular localization information and transcriptomics data [18, 37, 38], *C. zofingiensis* likely employs both PDHC and PDHC bypass routes, but mainly the former one, to supply acetyl-CoA in the chloroplast for fatty acid synthesis.

**Fig. 5:**
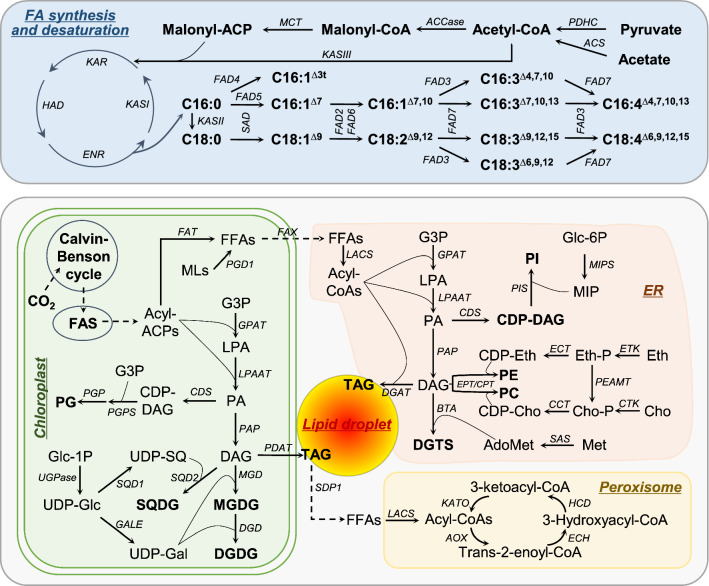
Lipid metabolic pathways in *C. zofingiensis.* ACCase, acetyl-CoA carboxylase; AdoMet, S-adenosylmethionine; AOX, acyl-CoA oxidase; BAT, betaine lipid synthase; CDS, phosphatidate cytidylyltransferase; CCT, choline-phosphate cytidylyltransferase; CHK, choline kinase; Cho, Choline; DAG, diacylglycerol; DGAT, Diacylglycerol acyltransferase; DGD, digalactosyldiacylglycerol synthase; DGDG, digalactosyl diacylglycerol; ECH, enoyl-CoA hydratase; ECT, CDP-Ethanolamine synthase; ENR, enoyl-ACP reductase; EPT/CPT, ethanolaminephosphotransferase/cholinephosphotransferase; Eth, Ethanolamine; ETK, ethanolamine kinase; GALE, UDP-galactose 4-epimerase; FAD, fatty acid desaturase; FAT, acyl-ACP thioesterase; G3P, glycerol-3-phosphate; GPAT, glycerol-3-phosphate acyltransferase; HAD, 3-ketoacyl-ACP dehydratase; HCD, 3-hydroxyacyl-CoA dehydrogenase; KAR, 3-ketoacyl-ACP reductase; KAS, 3-ketoacyl-ACP synthase; KATO, 3-ketoacyl-CoA thiolase; LACS, long-chain acyl-CoA synthetase; LPA, lysophosphatidic acid; LPAAT, lysophosphatidic acid acyltransferase; MCT, malonyl-CoA:acyl carrier protein transacylase; Met, methionine; MIPS, myo-inositol-1-phosphate synthase; MGD, monogalactosyldiacylglycerol synthase; MGDG, monogalactosyl diacylglycerol; MLDP, major lipid droplet protein; PA, phosphatidic acid; PAP, phosphatidate phosphatase; PC, phosphatidylcholine; PDAT, phospholipid:diacylglycerol acyltransferase; PE, phosphatidylethanolamine; PEAMT, phosphoethanolamine methyltransferase; PG, phosphatidylglycerol; PGP, phosphatidylglycerophosphatase; PGPS, phosphatidylglycerophosphate synthase; PI, phosphatidylinositol; PIS, phosphatidylinositol synthase; PGD1, Plastid Galactoglycerolipid Degradation1; SAD, stearoyl-ACP desaturase; SAS, S-adenosylmethionine synthase; SQDG, sulfoquinovosyl diacylglycerol; SDP1, Sugar-Dependent1 TAG lipase; TAG, triacyglycerol; UGPase, UDP-glucose pyrophosphorylase

De novo fatty acid synthesis in the chloroplast consists of a series of enzymatic steps mediated by acetyl-CoA carboxylase (ACCase), malonyl-CoA:acyl carrier protein (ACP) transacylase (MCT), and type II fatty acid synthase (FAS), an easily dissociable multisubunit complex (Fig. 5). The formation of malonyl-CoA from acetyl-CoA, a committed step in fatty acid synthesis, is catalyzed by ACCase [145]. The chloroplast-localized ACCase in *C. zofingiensis* is a tetrasubunit enzyme consisting of α-carboxyltransferase, β-carboxyltransferase, biotin carboxyl carrier protein, and biotin carboxylase. These subunits are well correlated at the transcriptional level [18, 33, 37, 39]. Malonyl-CoA has to be converted to malonyl-acyl carrier protein (ACP), through the action of MCT, before entering the subsequent condensation reactions for acyl chain extension. The condensation reactions are catalyzed by three types of 3-ketoacyl-ACP synthase (KAS): KAS III catalyzes the first condensation to form C4:0-ACP from malonyl-ACP and acetyl-CoA, KAS I catalyzes the subsequent condensation reactions up to C16:0-ACP, while KAS II catalyzes the formation of C18:0-ACP from C16:0-ACP. Following each condensation, additional reduction and dehydration steps are required to finish the two-carbon addition process, which are mediated in succession by 3-ketoacyl-ACP reductase (KAR), 3-hydroxyacyl-ACP dehydratase (HAD), and enoyl-ACP reductase (ENR) (Fig. 5). *C. zofingiensis* has been reported to possess one gene copy encoding the chloroplastic form of each KAS I, KAS II, KAS III, KAR, HAD and ENR; these genes are expressed in a well-coordinated manner to allow effective utilization of acetyl-CoA for the production of C16- and C18-ACPs [37].

Acyl-ACPs in the chloroplast can be either utilized by chloroplast-localized acyltransferases or converted to free fatty acids by the action of acyl-ACP thioesterase (FAT) [141]. Similar to *C. reinhardtii*, *C. zofingiensis* harbors a single-copy *FAT* gene, which correlates well with the de novo fatty acid synthetic genes at the transcriptional levels [18, 37]. The released free fatty acids, assisted with a fatty acid export 1 (FAX1), are translocated across chloroplast envelopes, which is characterized first in Arabidopsis [146] and then in algae [147, 148]. There are three putative FAX1-encoding genes present in *C. zofingiensis* [18]. Prior to integration into glycerolipids, the exported free fatty acids need to be ligated with CoA to form acyl-CoAs, catalyzed by long-chain acyl-CoA synthetase (LACS). Similar to vascular plants, such as Arabidopsis [149], algae possess multiple copies of putative *LACS* genes, e.g., three in *C. reinhardtii* [150], six in *C. zofingiensis* [151], five in *Phaeodactylum tricornutum* [152], and eight in *Thalassiosira pseudonana* [153]. Of the six *C. zofingiensis* LACS members, CzLACS2 through CzLACS5 are bona fide LACS enzymes and have overlapping yet distinct substrate preferences [151]. Considering the transcriptional expression data and subcellular localization results, CzLACS2 through CzLACS4, residing at endoplasmic reticulum (ER), are likely involved in TAG biosynthesis, while the peroxisome-localized CzLACS5 participates in fatty acid β-oxidation process [151].

In *C. zofingiensis*, unsaturated fatty acids dominate over saturated fatty acids (Fig. 4). The synthesis of unsaturated fatty acids involves a series of desaturases. Aside from the chloroplast-localized stearoyl-ACP desaturase (SAD) that is soluble and utilizes C18:0-ACP as substrate to form C18:1^∆9^-ACP [154], fatty acid desaturases (FADs) are usually membrane-bound and act on complex lipids for desaturation [141, 155]. *C. zofingiensis* contains two copies of *SAD* genes, of which *SAD1* has a much higher transcriptional level than *SAD2* and is considered as the major contributor of C18:1^∆9^ formation [18, 37]. In addition to C18:0-ACP, SAD1 accepts C16:0-ACP as the substrate for desaturation, yet in a considerably lower activity [156]. Other *C. zofingiensis* FADs include FAD2, FAD3, FAD4, FAD5, FAD6, FAD7 (Fig. 5) [37]. Both FAD2 and FAD6 are ω-6 desaturases: FAD2 is ER-localized and catalyzes desaturation at the ∆12 position of C18:1^∆9^, while FAD6 is chloroplast-localized and likely catalyzes desaturation at the ∆12 position of C18:1^∆9^ and ∆10 position of C16:1^∆7^ [141, 157]. FAD7, on the other hand, resides in the chloroplast envelop and likely accesses both extrachloroplastic and chloroplastic glycerolipids for the desaturation of C18:2^∆9,12^ and C18:3^∆6,9,12^ at their ∆15 position and of C16:2^∆7,10^ at its ∆13 position [158]. FAD4 and FAD5 are believed to act on the Δ3 position (trans) of C16:0 in PG and Δ7 position of C16:0 in MGDG, respectively [141]. Finally, FAD3 is likely to catalyze desaturation at the ∆4 position of C16 fatty acyls and ∆6 position of C18 fatty acyls [18]. The function of these membrane-bound FADs from *C. zofingiensis*, however, is awaiting experimental verification. Considering their transcriptional expression patterns and fatty acid changes upon stress conditions, these FADs may cooperate in a well manner and regulate desaturation degree of fatty acids in *C. zofingiensis* [18, 37].

Free fatty acids, on the other hand, can enter β-oxidation pathway for degradation. The location of fatty acid β-oxidation depends on organisms, e.g., peroxisomes for vascular plants and yeast, both peroxisomes and mitochondria for mammalian cells and probably microalgae [159]. Based on the study in *C. reinhardtii* [160], fatty acid β-oxidation in green microalgae is likely to occur in peroxisomes, similar to that in vascular plants [161]. Free fatty acids, once imported into peroxisomes, are converted to acyl-CoAs by peroxisome-localized LACS and then undergo oxidation via a cyclic reaction of four enzymatic steps: oxidation, hydration, dehydrogenation and thiolytic cleavage of an acyl-CoA. These steps involve acyl-CoA oxidase (AOX), enoyl-CoA hydratase (ECH), 3-hydroxyacyl-CoA dehydrogenase (HCD) and 3-ketoacyl-CoA thiolase (KATO) (Fig. 5). In *C. zofingiensis*, the four enzymes all have peroxisomal forms and their transcriptional expression tends to be down-regulated under several TAG inducing conditions [18, 37], suggesting fatty acid β-oxidation impairment contributes to TAG accumulation. *C. zofingiensis* has five isoforms of AOX and they may be functionally redundant, as is the case in *C. reinhardtii* [160]. A summary of genes involved in fatty acid biosynthesis, desaturation and β-oxidation in *C. zofingiensis* is listed in Table 2.

**Table 2:** Putative genes involved in fatty acid biosynthesis, desaturation and degradation in *C. zofingiensis*

Gene name abbreviations	Gene ID		Gene description	References
	JGI v5.2.3.2	GenBank		
*Acetyl-CoA production*				
PDHC E1A	Cz03g08090		Pyruvate dehydrogenase complex, E1 α-subunit	
PDHC E1B	Cz01g37230		Pyruvate dehydrogenase complex, E1 β-subunit	
ACS1	Cz09g15060	MK886788	Acetyl-CoA synthetase	[202]
ACS2	Cz12g10100	MK886789	Acetyl-CoA synthetase	[202]
De novo* fatty acid synthesis*				
α-CT	Cz02g12030		Carboxyltransferase α-subunit (ACCase complex)	
β-CT	Cz02g17060		Carboxyltransferase β-subunit (ACCase complex)	
BCCP	Cz03g28270		Biotin carboxyl carrier protein (ACCase complex)	
BC	Cz13g10110	GQ996717	Biotin carboxylase (ACCase complex)	[156]
MCT	Cz13g05150		Malonyl-CoA:acyl carrier protein transacylase	
KASI	Cz02g14160		3-ketoacyl-ACP synthase, I	
KASII	UNPLg00257		3-ketoacyl-ACP synthase, II	
KASIII	Cz18g03070		3-ketoacyl-ACP synthase, III	
KAR	Cz01g34370		3-ketoacyl-ACP reductase	
HAD	Cz01g09160		3-ketoacyl-ACP dehydratase	
ENR	Cz11g20040		Enoyl-ACP reductase	
*Fatty acid activation and export*				
FAT	Cz04g05080		Acyl-ACP thioesterase	
FAX1	Cz01g44210		Fatty acid export	
FAX2	Cz08g09020		Fatty acid export	
FAX3	Cz02g41140		Fatty acid export	
LACS1	Cz12g27140	MN317384	Long-chain acyl-CoA synthetase	[151]
LACS2	Cz11g20120	MN317385	Long-chain acyl-CoA synthetase; ER^a^	[151]
LACS3	Cz01g36150	MN317386	Long-chain acyl-CoA synthetase; ER^a^, LD^a^	[151]
LACS4	Cz07g22230	MN317387	Long-chain acyl-CoA synthetase; ER^a^, LD^a^	[151]
LACS6	Cz08g09130	MN317389	Long-chain acyl-CoA synthetase	[151]
*Fatty acid desaturation*				
SAD1	Cz04g09090	GQ996719	Stearoyl-ACP desaturase	[156]
SAD2	Cz13g17200		Stearoyl-ACP desaturase	
FAD6A	Cz08g04110		ω-6 fatty acid desaturase, chloroplastic type	
FAD6B	Cz11g21120		ω-6 fatty acid desaturase like, chloroplastic type	
FAD2	Cz03g33220		ω-6 fatty acid desaturase, ER type	
FAD7A	Cz04g31180		ω-3 fatty acid desaturase	
FAD7B	Cz06g28130		ω-3 fatty acid desaturase	
FAD5A	Cz07g00120		MGDG-specific palmitate ∆7 desaturase	
FAD5B	Cz06g00170		MGDG-specific palmitate ∆7 desaturase	
FAD5C	Cz13g01140		MGDG-specific palmitate ∆7 desaturase like	
FAD3A	Cz06g12050		∆4/∆6 desaturase like	
FAD3B	UNPLg00012		∆4/∆6 desaturase like	
FAD4	Cz12g10230		∆3 palmitate desaturase	
*Fatty acid β-oxidation*				
LACS5	Cz05g30060	MN317388	Long-chain acyl-CoA synthetase, peroxisome^a^	[151]
AOX1	Cz16g14110		Acyl-CoA oxidase	
AOX2	Cz07g30210		Acyl-CoA oxidase	
AOX3	Cz07g18040		Acyl-CoA oxidase	
AOX4	Cz08g04130		Acyl-CoA oxidase	
AOX5	Cz17g14150		Acyl-CoA oxidase	
ECH1	Cz16g07140		enoyl-CoA hydratase	
ECH2	Cz03g36260		enoyl-CoA hydratase	
ECH3	Cz04g19010		Enoyl-CoA hydratase/isomerase	
ECH4	Cz06g10230		Enoyl-CoA hydratase	
ECH5	Cz09g10030		Enoyl-CoA hydratase	
HCD	Cz11g22170		3-hydroxyacyl-CoA dehydrogenase	
KATO	Cz06g36270		3-ketoacyl-CoA thiolase	

^a^Where experimental evidence of a subcellular localization is available

### Membrane glycerolipid biosynthesis and turnover

The membrane glycerolipids in *C. zofingiensis* can be grouped into three categories: glycolipids (MGDG, DGDG and SQDG), phospholipids (PG, PC, PE and PI) and betaine lipid (DGTS) (Fig. 4). In general, the membrane glycerolipid metabolism in green algae is similar to that in vascular plants, except that green algae often contain DGTS and thus its metabolic pathway, while vascular plants lack it (Fig. 5) [162]. MGDG and DGDG, the major chloroplastic lipid fractions, are synthesized in the chloroplast. Using diacylglycerol (DAG) as the acceptor, the galactose moiety from UDP-galactose is transferred leading to MGDG formation, which is catalyzed by MGDG synthase (MGD). An additional transfer of the galactose moiety from UDP-galactose to MGDG, mediated by DGDG synthase (DGD), results in the formation of DGDG. SQDG, another chloroplastic lipid class that plays an important role in photosynthesis, is also biosynthesized in the chloroplast, which involves UDP-sulfoquinovose synthase (SQD1) and SQDG synthase (SQD2) that catalyze UDP-sulfoquinovose formation and transfer of sulfoquinovose from UDP-sulfoquinovose to DAG for SQDG synthesis, respectively [163]. Compared to *C. reinhardtii* that has only one gene copy for each *MGD*, *DGD*, *SQD1* and *SQD2* [164], *C. zofingiensis* harbors one copy for *MDG*, *SQD1* and *SQD2* each yet three copies for *DGD* [37]. Upon exposure of *C. zofingiensis* to stress conditions, MGDG, DGDG and SQDG all decreased, yet their biosynthetic pathways showed no transcriptional down-regulation [13, 17, 18, 37, 39].

Of the phospholipids, PG is believed to reside predominantly in the chloroplast and plays a role in photosystem II [165]. In addition, when subjected to sulfur deficient conditions, PG may accumulate and compensate for SQDG impairment to maintain photosystem I activity [166]. Unlike other chloroplastic membrane lipids, PG biosynthesis starts from cytidine diphosphate DAG (CDP-DAG), a product from the condensation of phosphatidic acid (PA) and cytidine triphosphate mediated by phosphatidate cytidylyltransferase (CDS). Through the action of phosphatidylglycerophosphate synthase (PGPS) on CDP-DAG and glycerol-3-phosphate (G3P), phosphatidylglycerophosphate is formed, which is further converted to PG by phosphatidylglycerophosphatase (PGP). *C. zofingiensis* is predicted to contain two *CDS* genes, one *PGPS* gene and one *PGP* gene [37]. Similarly, the transcriptional expression pattern of these genes is inconsistent with PG decrease observed under stress conditions [18, 37, 39]. PI also uses CDP-DAG as the precursor for synthesis, catalyzed by phosphatidylinositol synthase (PIS). There are two PIS-encoding genes present in *C. zofingiensis* [37]. Although *C. zofingiensis* harbors a gene encoding CDP-DAG-dependent phosphatidylserine (PS) synthase (PSS), no detectable level of PS is observed. This is probably due to that PS is rapidly converted to PE by PS decarboxylase (PSD), which is present in *C. zofingiensis* [37]. PE can also be synthesized from the CDP–ethanolamine pathway in which ethanolamine kinase (ETK), CDP–ethanolamine synthase (ECT) and ethanolaminephosphotransferase (EPT) are involved. PC, on the other hand, can be synthesized from the CDP–choline pathway and/or the methylation of PE; the former involves choline kinase (CHK), CDP–choline synthase (CCT) and cholinephosphotransferase (CPT) [167]. Similar to *Cyanidioschyzon merolae* and several *Chlamydomonas* species [168], *C. zofingiensis* possesses a single bifunctional EPT/CPT enzyme that is believed to catalyze the last biosynthetic step of both PE and PC [37]. As for DGTS, it is synthesized from DAG and *S*-adenosylmethionine by the action of DGTS synthase (BTA) [164]. Similar in *C. reinhardtii*, a single *BTA* gene is present in *C. zofingiensis*. Table 3 summarizes the putative genes involved in membrane glycerolipid biosynthesis in *C. zofingiensis*.

**Table 3:** Putative genes involved in membrane glycerolipid biosynthesis and turnover in *C. zofingiensis*

Gene name abbreviations	Gene ID		Gene description	References
	JGI v5.2.3.2	GenBank		
*Glycolipid synthesis (MGDG, DGDG and SQDG)*				
GALE1	Cz12g16020		UDP-galactose 4-epimerase	
GALE2	Cz08g10110		UDP-galactose 4-epimerase	
MGD	Cz08g30040		Monogalactosyldiacylglycerol synthase	
DGD1	Cz03g26070		Digalactosyldiacylglycerol synthase	
DGD2	Cz10g17090		Digalactosyldiacylglycerol synthase	
DGD3	Cz13g19030		Digalactosyldiacylglycerol synthase	
UGPase	UNPLg00641		UDP-glucose pyrophosphorylase	
SQD1	Cz03g31030		UDP-sulfoquinovose synthase	
SQD2	Cz07g23140		Sulfoquinovosyldiacylglycerol synthase	
*PG and PI synthesis*				
CDS1	Cz10g20080		Phosphatidate cytidylyltransferase	
CDS2	Cz01g36190		Phosphatidate cytidylyltransferase	
PGPS	Cz01g26070		Phosphatidylglycerophosphate synthase	
PGP	Cz12g07050		Phosphatidylglycerophosphatase	
MIPS	Cz01g18130		myo-inositol-1-phosphate synthase	
PIS1	Cz01g00060		phosphatidylinositol synthase	
PIS2	Cz17g13240		phosphatidylinositol synthase	
*DGTS, PC and PE synthesis*				
SAS1	Cz15g18200		S-adenosylmethionine synthase	
SAS2	Cz05g24030		S-adenosylmethionine synthase	
BTA	Cz01g13260		Betaine lipid synthase	
CHK	UNPLg00491		Choline kinase	
CCT	Cz12g21150		Choline-phosphate cytidylyltransferase	
EPT/CPT	Cz05g09130		Ethanolaminephosphotransferase/cholinephosphotransferase	
ETK	Cz11g15030		Ethanolamine kinase	
ECT	Cz05g17180		CDP-Ethanolamine synthase	
*Putative membrane lipid lipases*				
PGD1	Cz01g38020		Plastid Galactoglycerolipid Degradation1, Lipase class 3	
–	Cz02g15090		Alpha/beta hydrolase family	
–	Cz03g14190		Alpha/beta hydrolase family	
–	Cz01g06170		Lipase, SF153	[40]
–	Cz12g10010		Lipase, SF153	[40]

Considering that the decreases of membrane glycerolipids upon stress conditions are accompanied with no transcriptional down-regulation of their biosynthetic pathways [18, 37, 39], we hypothesize that their biosyntheses are maintained yet catabolic pathways mediated by lipases are likely stimulated leading to net decreases of these lipids. Microalgae harbor a number of genes encoding putative lipases, yet Plastid Galactoglycerolipid Degradation1 (PGD1) from *C. reinhardtii* is the only one that has been demonstrated to be involved in membrane lipid turnover [169]. This lipase, required for normal structure of thylakoid membranes, acts specifically on the *sn*-1 position of MGDG to release C18:1^∆9^ mainly for supporting TAG synthesis and is important during acclimation of *C. reinhardtii* to various adverse conditions [169, 170]. A single *PGD1* gene is present in the genome of *C. zofingiensis*, which shows a considerable up-regulation at the transcriptional level under multiple stress conditions, well consistent with the severe degradation of MGDG [18, 32, 37–39]. If *C. zofingiensis* PGD1 has the same function as its homolog in *C. reinhardtii*, which of course needs experimental verification, additional lipases are required to support the degradation of other chloroplastic lipids, such as DGDG, SQDG and PG. It has been suggested that Cz02g15090 and Cz03g14190 may encode such lipases as they cluster with *PGD1* based on the transcriptional expression pattern and are highly up-regulated under ND conditions [37]. Moreover, proteomics analysis of the LD fraction from *C. zofingiensis* has identified two lipases (Cz01g06170 and Cz12g10010), which are transcriptionally up-regulated upon ND and can enable yeast cells to produce more TAG when heterologously expressed, indicating that the two lipases may act on membrane lipids (of LDs and/or membrane contact sites between LDs and ER and between LDs and chloroplast) that they can access and contribute fatty acids to TAG synthesis [40]. Nevertheless, under SD and SS conditions that also cause severe degradation of chloroplastic lipids, the above mentioned four lipase genes exhibit no transcriptional up-regulation [18, 39]. Whether they are bona fide membrane lipid lipases and what lipid substrates they prefer are awaiting experimental evidences.

Interestingly, it has been reported that phospholipid:diacylglycerol acyltransferase (PDAT) from *C. reinhardtii*, in addition to functioning as an acyltransferase involved in TAG biosynthesis, has lipase activity toward a broad range of glycolipids and phospholipids, as suggested by the in vitro enzymatic assays [171]. Seemingly, PDAT in microalgae, transcriptionally up-regulated by ND, contributes to membrane lipid turnover in microalgae [171, 172], similar to the role of its homolog in vascular plants [173]. The gene encoding PDAT in *C. zofingiensis* is also up-regulated by ND as well as other stress conditions, yet the up-regulation extent is only moderate [18, 32, 37, 39], indicative of its mild contribution to membrane lipid turnover.

### TAG biosynthesis and degradation

In general, as in vascular plants, TAG biosynthesis in microalgae is believed to perform through two pathways, the acyl-CoA-dependent Kennedy pathway and the acyl-CoA-independent pathway [162]. The former pathway involves a series of enzymatic reactions catalyzed in succession by glycerol-3-phosphate acyltransferase (GPAT), 1-acyl-sn-glycerol-3-phosphate acyltransferase (LPAAT), phosphatidate phosphatase (PAP) and diacylglycerol acyltransferase (DGAT). GPAT mediates the first step of the acyl-CoA-dependent pathway leading to lysophosphatidic acid (LPA) formation by transferring the acyl moiety from an acyl-CoA to the *sn*-1 or *sn*-2 position of G3P [174]. Differing from vascular plants that harbor a high dose of GPAT isoforms [174], microalgae generally contain one chloroplastic form and one extrachloroplastic ER-localized form, which has been indicated in the green algae *C. reinhardtii* [141], *Monoraphidium neglectum* [175], *C. zofingiensis* [37] and *Lobosphaera incisa* [176], the heterokont algae *Nannochloropsis oceanica* [177] and *P. tricornutum* [178], and the red alga *Cyanidioschyzon merolae* [179]. In *C. zofingiensis*, the extrachloroplastic GPAT2 rather than the chloroplastic GPAT1 shows transcriptional up-regulation under multiple TAG inducing conditions and contributes to ND-associated TAG biosynthesis [18, 37, 39]. Similarly, it is believed that the extrachloroplastic GPAT (ER-localized) from *L. incisa* and *C. merolae* is involved in TAG biosynthesis [176, 179]. By contrast, in the diatom *P. tricornutum*, the chloroplastic GPAT seemingly plays a role in TAG synthesis, as suggested by its overexpression results [180]. The substrate preference of GPAT determines the fatty acid composition of *sn*-1 position of TAG. Considering that *C. zofingiensis* TAG *sn*-1/3 consists mainly of C18:1^∆9^ [17], GPAT2 may prefer C18:1^∆9^-CoA as the acyl donor.

LPAAT catalyzes the second acylation step by transferring the acyl moiety from an acyl-CoA to *sn*-2 position of LPA leading to PA formation. LPAAT also has both chloroplastic and extrachloroplastic forms in algae and the number varies depending on algal species [37, 141, 175, 177, 178]. It has been reported that the chloroplastic LPAAT of *C. reinhardtii* (CrLPAAT1), up-regulated by ND, prefers 16:0-CoA over C18:1^∆9^-CoA as the acyl donor for PA synthesis and is involved in TAG synthesis [181]. Consistent with the acyl-CoA preference of CrLPAAT1, overexpression of *CrLPAAT1* in *C. reinhardtii* promotes increase of TAG with *sn*-2 position being C16 acyls [181]. Interestingly, CrLPAAT2, an ER-localized chlorophyte-specific LPAAT enzyme, also prefers 16:0-CoA over C18:1^∆9^-CoA for PA formation, distinguishing from the canonical ER form of LPAAT that generally utilizes C18-CoAs as the acyl donor [182]. This is reasonable as *sn*-2 position of TAG in *C. reinhardtii* consists predominantly of C16:0 [183, 184]. By contrast, *C. zofingiensis* synthesizes TAG with *sn*-2 position mainly being C18:1^∆9^ [17]. These may reflect the great difference in acyl-CoA preference of LPAATs between the two closely related green algae *C. reinhardtii* and *C. zofingiensis*. There are three LPAAT isoforms in *C. zofingiensis*: LPAAT1 (homolog to CrLPAAT1), LPAAT2 (homolog to CrLPAAT2), and LPAAT3 [37]. As is the case in *C. reinhardtii*, both *C. zofingiensis LPAAT1* and *LPAAT2* genes are considerably up-regulated by ND, indicative of their involvement in TAG synthesis [37]. Whether the two LPAATs have substrate preference on C18-CoAs and to what extent they contribute to TAG synthesis are awaiting clarification via such experiments as in vitro enzymatic assays and in vivo functional characterization.

Prior to utilization for TAG synthesis, PA needs to be converted to DAG by the action of PAP. There is only one report about functional dissection of algal PAP, in which an extrachloroplastic PAP from *C. reinhardtii*, up-regulated transcriptionally by ND, contributes to TAG synthesis as suggested by both overexpression and suppression experiments [185]. *C. zofingiensis* harbors three putative PAP isoforms, one chloroplastic form (PAP1) and two extrachloroplastic forms (PAP2 and PAP3) [37]. Interestingly, these *PAP* genes respond differentially upon various stress conditions of ND, SD and SS: *PAP1* is up-regulated by ND, *PAP3* is up-regulated by SD and SS, while *PAP3* shows no up-regulation [18, 37, 39]. This indicates that *C. zofingiensis* may adopt different PAPs to cope with different stresses for TAG synthesis.

DGAT catalyzes the last and committed step in the Kennedy pathway for TAG synthesis by transferring the acyl moiety from an acyl-CoA to the *sn*-3 position of a DAG. There are three DGAT types, the membrane-bound type I (DGAT1) and type II (DGAT2 or DGTT) and the soluble type III (DGAT3) [186]. In general, microalgae harbor a much larger number of DGAT isoforms than vascular plants (e.g., one versus eleven for the type II), pointing to more complex regulations of microalgal TAG synthesis. Although why microalgae need such high dose of DGATs remains less understood, functional characterization of DGATs from multiple aspects has been conducted for many species including *C. reinhardtii* [183, 187, 188], *C. zofingiensis* [189–191], *H. pluvialis* [192, 193], *N. oceanica* [194–196], and *P. tricornutum* [197–199]. *C. zofingiensis* harbors ten putative DGAT isoforms, two type I (DGAT1A and DGAT1B) and eight type II (DGTT1 through DGTT8); all are predicted to be extrachloroplast-targeted [189]. For the transcriptional expression pattern upon ND, *DGAT1A*, *DGTT1*, *DGTT5*, *DGTT6* and *DGTT8* are considerably up-regulated, while the left five show slight or little variation [37, 189]. It is worth noting that not all ten DGAT isoforms have observed activity to restore TAG synthesis in a TAG-deficient yeast mutant [189, 190]. It seems not surprising as this phenomenon happens for other algae when expressing their *DGAT* genes in the same yeast mutant [183, 192–195, 199–201]. The functional failure of some putative algal DGATs in yeast may be attributed to (1) they are not bona fide DGAT enzymes, (2) their protein expression levels are too low to function or the expressed proteins are misassembled into nonfunctional forms in yeast, and (3) certain substrates or co-factors essential for the DGAT activity are absent from yeast, etc. Of the seven functional DGATs from *C. zofingiensis* based on the functional complementation results, DGAT1A has the highest activity followed by DGTT5, which is also supported by the in vitro DGAT assays using a wide range of substrates [189]. Clearly, DGAT1A and DGTT5, both residing at ER, have overlapping yet distinctive substrate specificity for both acyl-CoAs and DAGs: DGAT1A prefers eukaryotic DAGs with strong activity on C16:0- and C18:1^∆9^-CoAs, while DGTT5 prefers prokaryotic DAGs with weak activity on C16:0- and C18:1^∆9^-CoAs. Taken into account the transcriptional expression levels, functional complementation results in yeast, in vitro DGAT assays and the fatty acid composition in *sn*-2 and *sn*-1/3 positions of TAG [17, 189], DGAT1A likely contributes more than DGTT5 to ND-induced TAG in *C. zofingiensis*. Unlike ND, SD and SS stimulate the transcriptional expression of DGTT5 but not DGAT1A [18, 39]. This may partly explain why *C. zofingiensis* has a considerably higher TAG level under ND conditions as compared to under SD and SS conditions [17, 202] and further support the important role of DGAT1A in TAG synthesis. Interestingly, DGAT1A and DGTT5 possess strong activity on the CoA forms of ω-3 polyunsaturated fatty acids, such as eicosapentaenoyl-CoA (EPA-CoA) and docosahexaenoyl-CoA (DHA-CoA) [189]. In this context, *DGAT1A* and *DGTT5* have the potential to serve as promising gene targets of engineering for not only enhancing TAG production but also enriching ω-3 polyunsaturated fatty acids in TAG to add nutritional benefits.

The acyl-CoA-independent pathway for TAG synthesis is mediated by PDAT, which, differing from DGAT that uses acyl-CoAs, transfers the acyl from lipids (mainly the *sn*-2 position of phospholipids) to the *sn*-3 position of a DAG [203]. The enzyme has been named as PDAT, because the phospholipid PC was used as the acyl donor for investigating in vitro enzymatic activities in the pioneering study [204]. In fact, PDAT can utilize not only phospholipids but many other substrates as acyl donors, yet the activity and substrate preference are dependent on the PDAT sources [171, 204, 205]. Seemingly, PDAT functions more under non-stress than under stress conditions and its contribution to TAG synthesis is minor as compared to DGATs in *C. reinhardtii* [71, 171, 183]. In *C. zofingiensis*, *PDAT* is up-regulated under various TAG inducing conditions, yet in a less extent than *DGAT1A* and *DGTT5* [18, 37, 39], suggesting its minor contribution to TAG synthesis, as is the case in *C. reinhardtii*.

TAG accumulation is dependent on not only biosynthesis but also catabolism. Sugar-Dependent1 (SDP1) represents one of the most well studied TAG lipases, which was first characterized in Arabiodopsis [206]. This TAG lipase, similar to the yeast triacylglycerol lipase 3 and human adipose triglyceride lipase that harbor a patatin-like acyl-hydrolase domain, is LD-associated and acts mainly on TAG for releasing free fatty acids [206]. SDP1 homologs and their roles in TAG degradation have been reported in several microalgae including *P. tricornutum* [207], *L. incise* [208], *N. oceanica* [209] and *C. reinhardtii* [210]. *C. zofingiensis* contains a single SPD1-encoding gene, which is transcriptionally down-regulated under several TAG-inducing conditions [18, 37–39], suggesting the role of SDP1 in TAG breakdown in this alga as well. Moreover, in *C. zofingiensis*, another lipase (Cz02g29090) has a more severe down-regulation at its transcriptional level than SDP1 under stress conditions that induce TAG accumulation [18, 37, 39]. This lipase, homologous to AtLip1 from Arabidopsis with confirmed TAG lipase activity [211], is up-regulated upon removal of the stress that leads to TAG degradation [39]. In this context, Cz02g29090 may encode a TAG lipase and play a more important role than SDP1 in TAG catabolism in *C. zofingiensis*. Functional characterization of these lipases will help understand oleaginousness of this alga. The putative genes involved in TAG biosynthesis and catabolism in *C. zofingiensis* are listed in Table 4.

**Table 4:** Putative genes involved in TAG biosynthesis and degradation in *C. zofingiensis*

Gene name abbreviations	Gene ID		Gene description	References
	JGI v5.2.3.2	GenBank		
*G3P production*				
GPDH1	Cz12g24180		Glycerol-3-phosphate dehydrogenase	
GPDH2	Cz04g17090		Glycerol-3-phosphate dehydrogenase	
GPDH3	Cz10g29180		Glycerol-3-phosphate dehydrogenase	
GPDH4	Cz08g08240		Glycerol-3-phosphate dehydrogenase, mitochondrial form	
GK1	Cz12g27090		Glycerol kinase	
GK2	Cz18g13070		Glycerol kinase	
GK3	Cz05g35210		Glycerol kinase	
*TAG biosynthesis*				
GPAT1	Cz11g03260		Glycerol-3-phosphate acyltransferase, chloroplastic	[37]
GPAT2	Cz09g31330		Glycerol-3-phosphate acyltransferase	[37]
LPAAT1	Cz16g02090		1-acyl-sn-glycerol-3-phosphate acyltransferase, chloroplastic	
LPAAT2	Cz04g14150		1-acyl-sn-glycerol-3-phosphate acyltransferase	
LPAAT3	Cz10g20070		1-acyl-sn-glycerol-3-phosphate acyltransferase	
PAP1	Cz05g23060		Phosphatidate phosphatase, Lipin	
PAP2	Cz10g16040		Phosphatidate phosphatase	
PAP3	Cz16g11240		Phosphatidate phosphatase	
DGAT1A	Cz06g05010	MH523419	Diacylglycerol acyltransferase, type I; ER^a^	[189]
DGAT1B	Cz09g08290	MH523420	Diacylglycerol acyltransferase, type I	[189]
DGTT1	Cz06g35060	MH523421	Diacylglycerol acyltransferase, type II	[189]
DGTT2	Cz06g22030	MH523422	Diacylglycerol acyltransferase, type II	[189]
DGTT3	Cz09g23010	MH523423	Diacylglycerol acyltransferase, type II	[189]
DGTT4	Cz11g24150	MH523424	Diacylglycerol acyltransferase, type II	[189]
DGTT5	Cz09g27290	MH523425	Diacylglycerol acyltransferase, type II; ER^a^	[189]
DGTT6	Cz15g22140	MH523426	Diacylglycerol acyltransferase, type II	[189]
DGTT7	Cz11g21100	MH523427	Diacylglycerol acyltransferase, type II	[189]
DGTT8	Cz08g14220	MH523428	Diacylglycerol acyltransferase, type II	[189]
PDAT	Cz10g07180		Phospholipid:diacylglycerol acyltransferase	
*LD structural proteins*				
MLDP	Cz04g29220		Major lipid droplet protein; LD^a^	[40]
CLS1	Cz16g16140		Caleosin related protein; LD^a^	[40]
CLS2	Cz09g31050		Caleosin related protein; LD^a^	[40]
CLS3	Cz09g11210		Caleosin related protein; LD^a^	[40]
CLS4	Cz03g13150		Caleosin related protein; LD^a^	[40]
*Putative lipases involved in TAG degradation*				
SDP1	Cz05g29160		Sugar-Dependent1 TAG lipase	
–	Cz02g29090		Putative TAG lipase	

^a^Where experimental evidence of a subcellular localization is available

### Roles of LDs in TAG metabolism

As is the case in vascular plants, TAG, once synthesized, is packed into LDs for storage in algae [212]. LD is an organelle composed of an outer monolayer of polar lipids and a hydrophobic core filled with TAG and/or sterols; the outer monolayer is equipped with many proteins, such as structural proteins that maintain LD and functional enzymes [213]. In addition to serving as a reservoir for neutral lipids, LD is believed to play roles in many biological processes, such as lipid homeostasis, signaling, membrane trafficking, etc. [213–215]. Proteomic studies of LD fraction, which help understand LD biology and lipid metabolism, have been conducted for many algae including *C. reinhardtii* [216–218], *N. oceanica* [219], *Fistulifera* sp. [220], *Dunaliella bardawil* [221], *L. incise* [208], *P. tricornutum* [222], *C. zofingiensis* [40], and *Parachlorella kessleri* [223].

In *C. zofingiensis*, the LD fraction consists predominantly of TAG (over 90%), with a very low level of polar lipids [40]. The LD proteins can be classified mainly into functional unknown group, lipid metabolism, carbon metabolism and vesicle trafficking. Similar to in the other green algae, the most abundant LD protein in *C. zofingiensis* is the Major Lipid Droplet Protein (MLDP) [40], which is drastically up-regulated by stress conditions and correlates well with TAG accumulation [32, 37, 39]. MLDP, differing from oleosin, the major LD protein of vascular plants that possesses a long hydrophobic segment stretching into the TAG matrix of LDs [213], has no hydrophobic segment and resides on the surface of LD in a relatively loose association probably due to its intrinsic hydrophobic and topological properties [224, 225]. Expression of *C. zofingiensis MLDP* can restore the phenotypes (LD size and number and TAG content) of a *C. reinhardtii* mutant with insertional disruption in its *MLDP* gene and promote TAG content in a wild type *C. reinhardtii* strain [40], indicating that MLDP functions in not only maintaining LD but also facilitating TAG accumulation. Probably, *MLDP* overexpression facilitates sequestration of neutral lipids into LDs for storage, thus attenuating the end-product inhibition on TAG biosynthesis-related enzymes for improved TAG synthesis.

Intriguingly, many *C. zofingiensis* LD proteins have no homologs present in the LD proteome of *C. reinhardtii*, including certain functional unknown proteins, caleosins and lipases, suggesting the unique characteristic of *C. zofingiensis* LDs [40]. Caleosin harbors a central hydrophobic segment and thus can stretch into the mono-layer of LDs for anchoring [226]. Although widely present in LDs of vascular plants, caleosin represents a minor integral LD protein group and has an extremely lower abundance than oleosin [213]. By contrast, in *C. zofingiensis* LDs, caleosin proeins have comparable abundance to MLDP [40]. Unlike *MLDP* that is up-regulated at early stages of ND, caleosin genes are only up-regulated at late stages of ND. It is hypothesized that MLDP and caleosins have differential functions in LD biogenesis in *C. zofingiensis*: while MLDP is involved in formation and maintaining size of nascent LDs, caleosins probably function in fusing nascent LDs to large ones [40]. Moreover, a novel model has been proposed for *C. zofingiensis* LDs, which have connections with both the ER and chloroplast and are equipped with many structural proteins and functional enzymes: the structural proteins, such as MLDP, caleosins, and certain unknown proteins, are highly abundant and maintain the stability of LDs; by contrast, enzymes, such as polar lipid lipases and LACSs, collaborate with those ER and/or chloroplast-localized ones involved in lipid metabolism (e.g., GPAT, LPAAT, DGAT) to contribute to TAG biosynthesis [40]. It is worth noting that this study only performs a single time point proteomics analysis of LDs under ND conditions. The temporal dynamics of the LD proteome upon ND and differences in LD proteomes among various stress conditions, such as ND, SD, SS and HL, are interesting and remain to be further investigated.

### Mechanistic insights into lipid metabolism for TAG biosynthesis in C. zofingiensis

*C. zofingiensis* has the capacity to synthesize and accumulate high levels of TAG under various stress conditions, yet ND is the most efficient stimulus for triggering TAG accumulation [13, 17, 20, 32]. To understand the mechanisms of oleaginousness in *C. zofingiensis*, a multiomics study has been conducted, which involves a systematical and integrated analysis of time-resolved transcriptomes, lipidomes and metabolomes in response to ND [37]. The massive TAG accumulation in *C. zofingiensis* upon ND is attributed to coordinated regulation of multiple biological processes, including 1) stimulation of protein and amino acid catabolism, starch catabolism and glycolysis that allocate carbon flux to lipids, acetyl-CoA production via the PDHC and PDHC bypass pathways (providing precursor for de novo fatty acid synthesis), de novo fatty acid synthesis, fatty acid activation and desaturation and membrane lipid turnover (providing acyl-CoAs for TAG assembly), G3P production via the glycerol-3-phosphate dehydrogenase (GPHD)- and glycerol kinase (GK)-mediated pathways, acyltransferases (GPAT, LPAAT and DGAT) for TAG assembly, LD proteins, such as MLDP and caleosins, for LD formation and storage of TAG, ATP production via glycolysis and TCA cycle (providing energy molecules), NADPH production via the oxidative pentose phosphate (OPP) pathway and NADP^+^-dependent malic enzyme (ME) (providing reductants), and 2) suppression of TAG breakdown and fatty acid β-oxidation.

Compared to the green algae *C. reinhardtii* [227] and *M. neglectum* [175] with time-resolved transcriptomes under ND conditions, *C. zofingiensis* shows several key distinctions regarding oleaginousness for TAG accumulation [37]. First, unlike in *C. reinhardtii* or *M. neglectum* the PDHC bypass route contributes more than the chloroplastic PDHC route to acety-CoA production, the chloroplastic PDHC route serves as a major source of acety-CoA in *C. zofingiensis*. Second, regarding the genes involved in de novo fatty acid synthesis in response to ND, most show a well-coordinated up-regulation in *C. zofingiensis*; by contrast, many genes are down-regulated to different degrees in *C. reinhardtii* and *M. neglectum*. Third, in *C. zofingiensis* the ER-localized GPAT rather than the chloroplastic one contributes to ND-induced TAG synthesis, while in *C. reinhardtii* the chloroplastic GPAT likely contributes more than the ER one to TAG synthesis. Fourth, *C. zofingiensis* is superior to *C. reinhardtii* in the dose of DGAT isoforms and the abundance of their transcripts thus accumulates a considerably higher level of TAG. Fifth, while consisting of predominantly C16 fatty acyls in *C. reinhardtii*, the *sn*-2 position of TAG in *C. zofingiensis* is composed of mainly C18 fatty acyls, suggesting that *C. zofingiensis*, differing from *C. reinhardtii*, employs the eukaryotic pathway rather than the prokaryotic pathway as the major for TAG biosynthesis. Six, *C. reinhardtii* synthesizes a basal level of starch under favorable growth conditions and shows a transient increase of starch upon ND; by contrast, *C. zofingiensis* synthesizes starch constantly and the starch level decreases upon ND via stimulating starch degradation, providing carbon precursors for TAG synthesis.

*C. zofingiensis* accumulates TAG as the carbon and energy reservoir under stress conditions and when the carbon source is in excess, and obviously there are common attributes as well as distinctions in TAG metabolism among these different conditions [18, 33, 37–39, 228]. Nevertheless, how algal cells sense these conditions to trigger TAG synthesis and accumulation remains largely unknown and is worth of deep investigation.

## Carotenogenesis for astaxanthin biosynthesis in *C. zofingiensis*

**Fig. 6:**
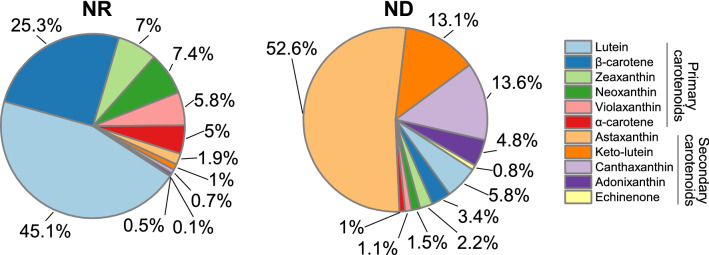
Profiles of carotenoids in *C. zofingiensis* under nitrogen replete (NR) and nitrogen deprivation (ND) conditions

**Fig. 7:**
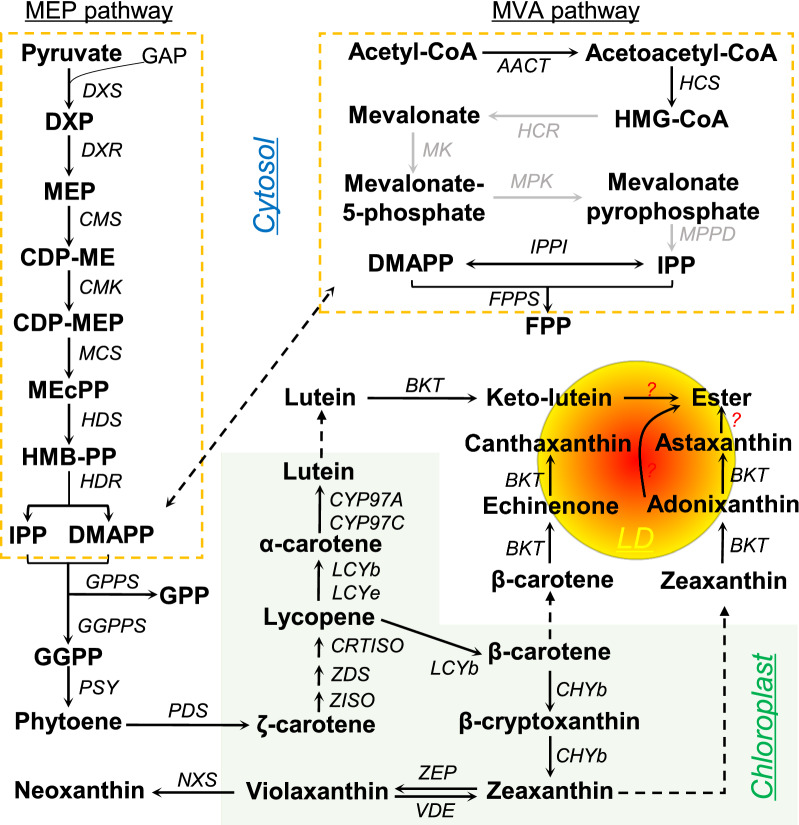
Carotenoid biosynthetic pathways in *C. zofingiensis.* AACT, acetoacetyl-CoA thiolase; AAT, long-chain-alcohol O-fatty-acyltransferase; BKT, beta-carotenoid ketolase; CDP-ME, 4-diphosphocytidyl-2-C-methylerythritol; CDP-MEP, 4-diphosphocytidyl-2-C-methyl-D-erythritol 2-phosphate; CHYb, beta-carotenoid hydroxylase; CMK, 4-diphosphocytidyl-2-C-methyl-D-erythritol kinase; CMS, 2-C-methyl-D-erythritol 4-phosphate cytidylyltransferase; CRTISO, carotenoid isomerase; CYP97A, cytochrome P450 beta hydroxylase; CYP97C, cytochrome P450 epsilon hydroxylase; DMAPP, dimethylallyl pyrophosphate; DXR, 1-deoxy-D-xylulose 5-phosphate reductoisomerase; DXP, 1-deoxy-D-xylulose 5-phosphate; DXS, 1-deoxy-D-xylulose 5-phosphate synthase; FPP,farnesyl diphosphate; FPPS, farnesyl diphosphate synthase; GAP, glyceraldehyde 3-phosphate; GGPP, geranylgeranyl diphosphate; GGPPS, geranylgeranyl diphosphate synthase; GPP, geranyl diphosphate; GPPS, geranyl diphosphate synthase; HCS, hydroxymethylglutaryl-CoA synthase; HDR, 4-hydroxy-3-methylbut-2-en-1-yl diphosphate reductase; HDS, 4-hydroxy-3-methylbut-2-en-1-yl diphosphate synthase; HGM-CoA, 3-hydroxy-3-methylglutaryl-CoA; HMB-PP, (E)-4-Hydroxy-3-methyl-but-2-enyl pyrophosphate; IPP, isopentenyl pyrophosphate; IPPI, isopentenyl-diphosphate Delta-isomerase; LCYb, lycopene beta cyclase; LCYe, lycopene epsilon cyclase; LD, lipid droplet; MCS, 2-C-methyl-D-erythritol 2,4-cyclodiphosphate synthase; MEcPP, 2-C-methyl-D-erythritol 2,4-cyclodiphosphate; MEP, 2-C-methylerythritol 4-phosphate; NXS, neoxanthin synthase; PDS, phytoene desaturase; PSY, phytoene synthase; VDE, violaxanthin de-epoxidase; ZDS, zeta-carotene desaturase; ZEP, zeaxanthin epoxidase; ZISO, zeta-carotene isomerase

The carotenoid profile in *C. zofingiensis* has been reported by many independent research groups and varies likely due to the use of different culture conditions and analytic methods [22, 24, 32, 33, 41, 54, 55, 104, 107]. In general, *C. zofingiensis* contains predominantly primary carotenoids including lutein, β-carotene, zeaxanthin, neoxanthin, violaxanthin, and α-carotene under favorable growth conditions, with lutein and β-carotene being the major ones; upon stress conditions, such as ND, secondary carotenoids including astaxanthin, canthaxanthin, keto-lutein, echinenone, and adonixanthin accumulate and become the dominated portion of carotenoids (Fig. 6). Nevertheless, the astaxathin content in *C. zofingiensis*, ranging from 0.1 to 1% of dry weight depending on culture conditions (Table 1), is much lower than that in *H. pluvialis* (4% of dry weight). This necessitates the requirements of complicated downstream purification processes for *C. zofingiensis* astaxanthin, leading to input of more production costs and thus the impairment of commercial potential. Genetic engineering of *C. zofingiensis* may have the potential to break the inherent constraints on astaxanthin accumulation, which relies on a better understanding of carotenogenesis for astaxanthin biosynthesis in this alga. With the assistance of whole genome sequence and reconstruction of carotenogenic pathways [33, 41], carotenogenic genes for synthesis of the carotenoid precursors isopentenyl pyrophosphate (IPP) and dimethylallyl pyrophosphate (DMAPP), of primary carotenoids from IPP/DMAPP, and of astaxanthin from β-carotene have been identified (Fig. 7 and Table 5), which are detailed in the subsequent sections.

**Table 5:** Putative genes involved in carotenoid biosynthesis in *C. zofingiensis*

Gene name abbreviations	Gene ID		Gene description	References
	JGI v5.2.3.2	GenBank		
*IPP/DMAPP synthesis*				
DXS	Cz02g35280	MT188717	1-deoxy-D-xylulose 5-phosphate synthase	[41]
DXR	Cz07g19130	MT188718	1-deoxy-D-xylulose 5-phosphate reductoisomerase	[41]
CMS	Cz12g10090	MT188719	2-C-methyl-D-erythritol 4-phosphate cytidylyltransferase	[41]
CMK	Cz11g24270	MT188720	4-diphosphocytidyl-2-C-methyl-D-erythritol kinase	[41]
MCS	Cz02g10180	MT188721	2-C-methyl-D-erythritol 2,4-cyclodiphosphate synthase	[41]
HDS	Cz13g08020	MT188722	4-hydroxy-3-methylbut-2-en-1-yl diphosphate synthase	[41]
HDR	Cz05g23010	MT188723	4-hydroxy-3-methylbut-2-enyl diphosphate reductase	[41]
AACT	Cz04g25080	MT188724	Acetoacetyl-CoA thiolase	[41]
HCS	Cz19g02060	MT188725	Hydroxymethylglutaryl-CoA synthase	[41]
IPPI1	Cz03g02150		Isopentenyl-diphosphate delta-isomerase	[41]
IPPI2	Cz08g23110		Isopentenyl-diphosphate delta-isomerase	[41]
IPPI3	Cz03g10110		Isopentenyl-diphosphate delta-isomerase	[41]
*IPP/DMAPP to carotenoids*				
GPPS	Cz02g31110		Geranyl diphosphate synthase	
FPPS	Cz01g03190		Farnesyl diphosphate synthase	
GGPPS	Cz02g19200		Geranylgeranyl diphosphate synthase	
PSY	Cz05g32220	FR670783	Phytoene synthase	[239]
PDS	Cz02g32280	EF621406	Phytoene desaturase	[245]
ZDS	Cz10g17010		ζ-carotene desaturase	
ZISO	Cz10g17130		ζ-carotene isomerase	
CRTISO1	Cz16g01210		Carotenoid isomerase	
CRTISO2	Cz12g03260		Carotenoid isomerase	
CRTISO3	Cz14g22040		Carotenoid isomerase	
LCYe	Cz09g18310	HE664109	Lycopene ε-cyclase	[250]
LCYb	Cz12g10170	FN563998	lycopene β-cyclase	[251]
CYP97A1	Cz13g16110		Cytochrome P450 β-hydroxylase	
CYP97A2	Cz09g14130		Cytochrome P450 β-hydroxylase	
CYP97C	Cz09g07100		Cytochrome P450 ε-hydroxylase	
CHYb	Cz12g16080	EU016205	β-carotenoid hydroxylase	[304]
BKT1	Cz13g13100	AY772713	β-carotenoid ketolase/oxygenase	[260]
BKT2	Cz04g11250		β-carotenoid ketolase/oxygenase	
ZEP	Cz07g30060	HE863825	Zeaxanthin epoxidase	[252]
VDE	Cz06g02070		Violaxanthin de-epoxidase	
NXS	Cz15g04070		Neoxanthin synthase	

### IPP/DMAPP formation

There are two pathways for the biosynthesis of IPP/DMAPP in vascular plants, the 2-C-methylerythritol 4-phosphate (MEP) pathway and mevalonate (MVA) pathway [229]. The MEP pathway occurs in the chloroplast and converts pyruvate and glyceraldehyde 3-phosphate (GAP) to IPP/DMAPP via the intermediates 1-deoxy-d-xylulose 5-phosphate (DXP), MEP, 4-diphosphocytidyl-2-C-methylerythritol (CDP-ME), 4-diphosphocytidyl-2-C-methyl-d-erythritol 2-phosphate (CDP-MEP), 2-C-methyl-d-erythritol 2,4-cyclodiphosphate (MEcPP), and (E)-4-Hydroxy-3-methyl-but-2-enyl pyrophosphate (HMB-PP), catalyzed in order by DXP synthase (DXS), DXP reductoisomerase (DXR), 2-C-methyl-d-erythritol 4-phosphate cytidylyltransferase (CMS), 4-diphosphocytidyl-2-C-methyl-d-erythritol kinase (CMK), 2-C-methyl-d-erythritol 2,4-cyclodiphosphate synthase (MCS), 4-hydroxy-3-methylbut-2-en-1-yl diphosphate synthase (HDS), and 4-hydroxy-3-methylbut-2-en-1-yl diphosphate reductase (HDR). By contrast, the MVA pathway occurs in the cytosol and starts with acetyl-CoA for producing IPP/DMAPP via the intermediates acetoacetyl-CoA, 3-hydroxy-3-methylglutaryl-CoA (HGM-CoA), mevalonate, mevalonate-5-phosphate, and mevalonate pyrophosphate, catalyzed successively by acetoacetyl-CoA thiolase (AACT), hydroxymethylglutaryl-CoA synthase (HCS), HMG-CoA reductase (HCR), mevalonate-5-kinase (MK), phosphomevalonate kinase (MPK), and mevalonate-5-pyrophosphate decarboxylase (MPPD). IPP and DMAPP can be interconverted by the action of IPP delta-isomerase (IPPI).

All enzymes involved in the MEP pathway have been identified in *C. zofingiensis* and each are encoded by a sing-copy gene; by contrast, many enzymes involved in the MVA pathway are missing (Fig. 7 and Table 5). Similarly, the MVA pathway is also incomplete in the green algae *C. reinhardtii* and *H. pluvialis* [230, 231], suggesting that it may be lost during the evolution of green algae [232]. Moreover, it is believed that *C. reinhardtii* and *H. pluvialis* utilize the MEP pathway rather than the MVA pathway to supply IPP/DMAPP for carotenoid biosynthesis [231, 233]. Fosmidomycin and mevinolin are inhibitors targeting the MEP pathway and the MVA pathway, respectively. Carotenoid levels in *C. zofingiensis* were impaired by fosmidomycin instead of mevinolin, indicating that this alga also employs the MEP pathway for carotenoid biosynthesis [14]. Intriguingly, upon ND or SD that triggers accumulation of secondary carotenoids including astaxanthin, the MEP pathway was not up-regulated at the transcriptional level in *C. zofingiensis* [39, 41]. Probably, no up-regulation of the MEP pathway is needed to provide precursors for carotenoids, as the level of total carotenoids in *C. zofingiensis* shows little change. By contrast, in *H. pluvialis* the MEP pathway showed a considerable up-regulation in response to ND and/or HL [230, 234]. This difference may partially explain why *C. zofingiensis* synthesizes a lower level of astaxanthin than *H. pluvialis*.

### Biosynthesis of primary carotenoids

Condensation of one DMAPP with one, two and three IPP molecules produces geranyl diphosphate (GPP), farnesyl diphosphate (FPP) and geranylgeranyl diphosphate (GGPP), which are catalyzed by GPP synthase (GPPS), FPP synthase (FPPS) and GGPP synthase (GGPPS), respectively. GGPP is the direct metabolic precursor for carotenoids. The head-to-head condensation of two GGPP molecules mediated by phytoene synthase (PSY) leads to formation of phytoene, a colorless C40 carotenoid. Phytoene is then converted to lycopene through several desaturation and isomerization steps catalyzed by phytoene desaturase (PDS), ζ-carotene isomerase (ZISO), ζ-carotene desaturase (ZDS) and carotenoid isomerase (CRTISO) (Fig. 7). It is worth noting that some photosynthetic bacteria, differing from vascular plants and eukaryotic algae, employ a single enzyme, crtI, to catalyze the formation of lycopene from phytoene [235–237]. *C. zofingiensis* harbors a single gene for each of GPPS, FPPS, GGPPS, PSY, PDS, ZISO and ZDS and three gene copies for CRTISO (Table 5). PSY is considered as the first and key rate-limiting enzyme that determines the metabolic flux to carotenoids [238]. Heterologous expression of the *C. zofingiensis PSY* gene in *C. reinhardtii* led to increased level of carotenoids [239], consistent with previous studies of overexpressing *PSY* gene in algae and vascular plants [238, 240, 241]. PDS that catalyzes the desaturation of phytoene to ζ-carotene is also considered as a rate-limiting enzyme for carotenoid biosynthesis [242, 243]. In *C. zofingiensis*, *PDS* was up-regulated under carotenogenic conditions of HL and correlated with carotenoid accumulation [244, 245]. The up-regulation of *PDS* by HL also occurs in *H. pluvialis*, not only at the transcriptional level but also at the translational level [246]. It has been reported that overexpression of *PDS* promoted carotenoid synthesis in several algae including *C. zofingiensis* [34], *H. pluvialis* [243] and *C. reinhardtii* [247]. Besides, PDS mutants with certain point mutations showed strong resistance to the herbicide norflurazon and can be used as dominant selectable marker for algal transformation [34, 243, 247, 248]. Interestingly, the mutation of L (leucine) to F (phenylalanine) at the position 516 of *C. zofingiensis* PDS, unlike other mutations that confer norflurazon resistance yet attenuate desaturation activity, enhanced the desaturation activity by 30% [249].

The cyclization of lycopene is critical as it determines the destination of lycopene to either β-carotene or α-carotene and their downstream derivatives. The action of lycopene β-cyclase (LCYb) adds β-ionone rings on both ends of lycopene leading to β-carotene formation, while the collaboration of LCYb and lycopene ε-cyclase (LCYe) generates a β-ionone ring on one end and a ε-ionone ring on the other end resulting in α-carotene formation (Fig. 7). *C. zofingiensis* harbors a single gene for each of LCYb and LCYe; LCYb can convert lycopene and δ-carotene to β-carotene and α-carotene, respectively, while LCYe only acts on lycopene to produce δ-carotene [250, 251]. These two genes have differential expression patterns, e.g., *LCYb* is up-regulated, while *LCYe* is considerably down-regulated in response to stress conditions that trigger accumulation of β-carotene derivatives at the expense of α-carotene derivatives [18, 32, 39, 250, 252], supporting the determining roles of LCYb and LCYe in allocating carotenoid flux between the two branching ways.

Catalyzed by the non-heme di-iron type of β-carotenoid hydroxylase (CHYb), β-carotene undergoes two sequential hydroxylation steps leading to zeaxanthin formation via the intermediate β-cryptoxanthin. By contrast, the hydroxylation of α-carotene to lutein is mediated by the heme-containing cytochrome P450 enzymes CYP97A and CYP97C, which add a hydroxyl group on the β- and ε-rings of α-carotene, respectively (Fig. 7). Of the four hydroxylase genes in *C. zofingiensis* (one CHYb gene, two CYP97A genes and one CYP97C genes), only *CHYb* has been functionally characterized [14, 253]. Unlike *CHYb* that varies in its expression pattern depending on stress conditions, CYP97A and CYP97C genes are normally down-regulated, in support of attenuated lutein accumulation in *C. zofingiensis* [18, 32, 39, 244]. Zeaxanthin, by the action of zeaxanthin epoxidase (ZEP) that introduces epoxy groups, is converted to violaxanthin via the intermediate antheraxanthin. Violaxanthin can also be converted back to zeaxanthin by violaxanthin de-epoxidase (VDE). The interconversion between zeaxanthin and violaxanthin is referred to as the violaxanthin cycle, which is widely present in vascular plants and algae and plays important roles in photoprotection against adverse environments [254, 255]. Moreover, the introduction of an allenic double bond to violaxanthin generates neoxanthin, which is mediated by neoxanthin synthase (NXS). There is a single gene present in *C. zofingiensis* encoding for each of ZEP, VDE and NXS (Table 5). These genes tend to undergo transcriptional suppression upon stress conditions, consistent with the impaired synthesis of violaxanthin and neoxanthin [18, 32, 39].

### Astaxanthin biosynthesis

Unlike the primary carotenoids mentioned above, astaxanthin is a keto-carotenoid and its formation requires additional ketolation steps mediated by β-carotenoid ketolase (BKT) in algae [111, 256]. The biosynthesis of astaxanthin from β-carotene, involving two hydroxylation steps and two ketolation steps in total, has multiple routes and may vary in different organisms. Considering that BKT is efficient in converting β-carotene to canthaxanthin but poor in converting zeaxanthin to astaxanthin and CHYb has strong activity to catalyze astaxanthin formation from canthaxanthin [253, 257–259], *H. pluvialis* is likely to employ the route with two stepwise ketolation reactions followed by two stepwise hydroxylation reactions as the major contributor for astaxanthin synthesis. *C. zofingiensis* is predicted to contain two *BKT* genes, *BKT1* and *BKT2*, yet only *BKT1* has been functionally characterized [228, 260]. Intriguingly, inactivation of *BKT1* led to complete abolishment of astaxanthin accumulation [33, 261], indicating that *BKT1* instead of *BKT2* is involved in astaxanthin biosynthesis in *C. zofingiensis*. Differing from *H. pluvialis* that contains only a trace amount of canthaxanthin, *C. zofingiensis* accumulates canthaxanthin up to 30% of the secondary carotenoids [13, 19, 22, 54, 55], indicating that its CHYb may have no or low activity in converting canthaxanthin to astaxanthin thus leading to the buildup of canthaxanthin as an end product. On the other hand, *C. zofingiensis* synthesizes adonixanthin, the intermediate of ketolating zeaxanthin to astaxanthin that is not detectable in *H. pluvialis*, and adonixanthin is stimulated to accumulate upon astaxanthin-inducing conditions [18, 19, 32, 39]. Moreover, suppression of BKT activity by the specific chemical inhibitor diphenylamine or *BKT1* mutation boosts zeaxanthin accumulation at the expense of astaxanthin [33, 261, 262]. These results, plus the functional validation of *BKT1* in a zeaxanthin-producing *E. coli* system [263], suggesting that the *C. zofingiensis* BKT accepts zeaxanthin as the substrate to form astaxanthin with a moderate efficiency. In line with these studies, the in vitro assays of *C. zofingiensis* BKT and CHYb provided solid evidence to support that BKT1 is able to ketolating zeaxanthin to astaxanthin, while CHYb has no activity in hydroxylating canthaxanthin to astaxanthin [14]. In this context, *C. zofingiensis* employs a route different from *H. pluvialis* for astaxanthin synthesis, namely, the CHYb-catalyzed hydroxylation of β-carotene to zeaxanthin first and then the BKT-catalyzed ketolation of zeaxanthin to astaxanthin (Fig. 7). It is worth noting that *C. zofingiensis* BKT1 may also act on lutein and adds a keto group on the β-ring to generate keto-lutein, as *BKT1* dysfunction impairs keto-lutein accumulation [261]. By contrast, no keto-lutein is detected in *H. pluvialis*.

The amino acid variance in certain positions of the BKT polypeptides may cause the functional difference of BKT enzymes between *C. zofingiensis* and *H. pluvialis*. It has been reported that singe-amino acid mutations in over ten positions of *C. zofingiensis* BKT1 abolished astaxanthin accumulation [33, 36, 261]. One of these mutated positions, R51 (arginine at the position 51), may be critical for *C. zofingiensis* BKT1 in the function of ketolating zeaxanthin to astaxnathin [14]. First, in the corresponding position of *H. pluvialis* BKT that has no activity on zeaxanthin ketolation, the amino acid residue is lysine (K), different from *C. zofingiensis* BKT1. Second, substitution of R51 with K in *C. zofingiensis* BKT1 blocks astaxanthin accumulation and promotes zeaxanthin level considerably [36]. Third, the BKT from *C. reinhardtii*, which resembles *C. zofingiensis* BKT1 and functions in converting zeaxanthin to astaxanthin [263], harbors the amino acid residue R in the position corresponding to R51 of *C. zofingiensis* BKT1. Moreover, in this position, *C. zofingiensis* BKT2 that is believed to have no activity on zeaxanthin contains the same amino acid residue K as *H. pluvialis* BKT. Functional validation of the K-to-R mutant of *H. pluvialis* BKT remains to be performed and would provide insights into understanding the substrate utilization of BKT enzymes for zeaxanthin. The functional difference of CHYb enzymes between *C. zofingiensis* and *H. pluvialis* may be also attributed to the amino acid variance in their polypeptides. It has been reported that overexpression of *CrBKT* in *C. reinhardtii* and the vascular plants *Arabidopsis thaliana* and *Lycopersicon esculentum* each results in the accumulation of a substantial amount of canthaxanthin [253, 263, 264]. Therefore, the endogenous CHYb enzymes from these organisms are likely similar to *C. zofingiensis* CHYb and have no/low activity in converting canthaxanthin to astaxanthin. In silico analysis of these CHYb polypeptides with *H. pluvialis* CHYb has suggested involvement of several candidate amino acid residues in the function of ketolating canthaxanthin to astaxanthin [14].

Similar in *H. pluvialis*, astaxanthin in *C. zofingiensis* is stored in cytoplasmic LDs [14, 109, 265]. As the primary carotenoids including lycopene, β-carotene and zeaxanthin are synthesized in the chloroplast, whereas the ketolation steps for astaxanthin biosynthesis occur outside of the chloroplast [108], certain carotenoids have to transport across the chloroplast envelops for supporting extrachloroplastic astaxanthin synthesis. It is believed that in *H. pluvialis* the transport takes place after β-ionone ring cyclization, namely, β-carotene is the intermediate exported from the chloroplast during astaxanthin induction [109]. The exported β-carotene is likely packed into cytoplasmic LDs and undergoes ketolation and hydroxylation steps for astaxanthin biosynthesis, considering that both activities of BKT and CHYb are detected in the isolated LD fractions [108]. This may not hold true in *C. zofingiensis*, as neither BKT nor CHYb is present in LDs based on the proteomics analysis of the purified LD fraction [40]. Albeit lacking experimental evidence, *C. zofingiensis* BKT and CHYb are predicted to reside in the ER and chloroplast, respectively [41]. In this context, export of both β-carotene and zeaxanthin from the chloroplast is in need to support the BKT-mediated ketolation for producing canthaxanthin and astaxanthin, respectively. Nevertheless, if the CHYb activity is also present outside the chloroplast in *C. zofingiensis*, zeaxanthin export may be not necessary. As no signs of vesicular transport observed, it has been hypothesized that carotenoid binding proteins rather than vesicular transport are involved in facilitating export of β-carotene in *H. pluvialis* [109, 246]. Nevertheless, no such protein has so far been identified. In algae under stress conditions, LDs are connected with both the chloroplast and ER and may serve as bridges to allow diffusion of lipids, such as DAG between the chloroplast and ER along the LD-delimiting mono-layer [266]. This may also be applicable to carotenoids in *C. zofingiensis*, for example, β-carotene and zeaxanthin are translocated along the LD mono-layer to ER for ketolation mediated by BKT; the ketolated carotenoids, such as astaxanthin, canthaxanthin and adonixanthin, can diffuse as well along the LD mono-layer and enter LDs for storage.

### Esterification of astaxanthin

Astaxanthin in *C. zofingiensis* and *H. pluvialis* has long been found to be present mainly in the form of ester (mono-ester and di-ester), which reaches up to 90% of total astaxanthin depending on algal strains and culture conditions [13, 14, 16, 88, 267]. It is thought that the formation of astaxanthin ester from free astaxanthin involves acyltransferase(s) that may transfer an acyl moiety from acyl-CoA and/or acyl-containing lipids to the hydroxyl end groups of astaxanthin. Nevertheless, the enzyme(s) responsible for esterification of astaxanthin have yet to be identified, albeit there have been several presumptions. In mammals, DGAT1, besides the involvement in TAG synthesis, has been demonstrated to also possess acyl CoA:retinol acyltransferase activity and catalyze retinol esterification [268, 269]. Retinol is a degradation production of carotenoids, raising the hypothesis whether DGAT(s) have the ability to esterify astaxanthin. Based on the results that the ER fraction (where DGAT enzymes reside) can mediate astaxanthin ester synthesis by feeding β-carotene in vitro and the addition of DGAT inhibitors impair astaxanthin ester formation, DGATs have been proposed as the candidate enzymes responsible for astaxanthin esterification in *H. pluvialis* [15]. However, it cannot be excluded that unknown acyltransferases that have astaxanthin esterification activity may also be present in the ER fraction and vulnerable to DGAT inhibitors. It is worth noting that in *C. reinhardtii* and some vascular plants, albeit multiple *DGATs* are present in their genomes, the reconstruction of astaxanthin biosynthesis pathways in them leads to the accumulation of free astaxanthin rather than ester [263, 264, 270, 271], questioning the role of DGATs in astaxanthin esterification. Since both *C. zofingiensis* and *H. pluvialis* harbor multiple *DGAT* gene copies yet lack well-established genetic tools [189, 192, 193], it is challenging to validate the esterification function of these *DGAT* genes in vivo via genetic manipulations. Functional analysis in free astaxanthin-producing yeast may represent an option. Recently, heterologous expression of the ten *C. zofingiensis DGAT* genes each in a free astaxanthin-producing yeast strain has been conducted and the results failed to support the role of DGATs in astaxanthin esterification [14]. Another proposed candidate enzyme for astaxanthin esterification is a long-chain-alcohol O-fatty-acyltransferase from *C. zofingiensis* [33], which is transcriptionally up-regulated under many astaxanthin inducing conditions [14, 32]. Nevertheless, heterologous expression of this gene in the free astaxanthin-producing yeast strain also failed to produce detectable esterified astaxanthin [14].

Moreover, it has been reported in vascular plants that esterase-like enzymes are involved in esterification of several carotenoids. One is PYP1, an esterase/lipase/thioesterase family of acyltransferase from tomato that contributes to esterification of violaxanthin and neoxanthin [272]. The other one is XAT, a Gly-Asp-Ser-Leu motif-containing esterase/lipase, which has the ability to esterify lutein, zeaxanthin and cryptoxanthin using a broad range of acyl donors [273]. They may have the potential to also function as astaxanthin esterase. Searching *C. zofingiensis* genome reveals the presence of homolog of PYP1 (encoded by Cz02g16070) but not of XAN. Experimental evidence is needed for clarifying function of this PYP1 homolog. On the other hand, a high-throughput forward genetic screening via random mutagenesis represents an alternative option to probe the genuine acyltransferase responsible for astaxanthin esterification. Although labor-intensive and time-consuming, it has been successfully applied to *C. zofingiensis* for identifying genes involved in astaxanthin biosynthesis and lipid metabolism [33, 35, 36, 261].

Interestingly, it has been reported that some of the astaxanthin-producing algae accumulate only free astaxanthin [105, 122], raising the questions that why *C. zofingiensis* and *H. pluvialis* synthesize predominantly esterified astaxanthin, whereas some algae produce only free astaxanthin and what’s the biological significance of astaxanthin esterification. Identification and characterization of the genuine astaxanthin esterase and mutants of algae defective in this enzyme would help address these questions.

### Mechanistic insights into carotenogenesis for astaxanthin biosynthesis in C. zofingiensis

As mentioned above, *C. zofingiensis* and *H. pluvialis* tend to synthesize and accumulate astaxanthin under stress conditions. It is widely accepted that astaxanthin formation is a survival strategy of algae to cope with adverse conditions [56, 111]. Astaxanthin biosynthesis may offer multiple layers of protection to *C. zofingiensis* cells. First, astaxanthin accumulates in cytoplasmic LDs that reside peripherally and surround the chloroplast [14, 55]. These astaxanthin-containing LDs may function like a sunscreen to reduce the amount of light impinging on the chloroplast and other organelles, thus attenuating photosynthetic photoinhibition and photodamage associated with excess photons. Second, *C. zofingiensis* accumulates reactive oxygen species (ROS) triggered by stress conditions [13, 244]; astaxanthin has strong antioxidation activity and can serve as a powerful scavenger to mitigate excess ROS for preventing algal cells from damage. Third, astaxanthin is more abundant in oxygen content than other carotenoids in *C. zofingiensis*; astaxanthin buildup has the potential to lower intracellular oxygen levels and thus the generation of ROS.

Exposure of *C. zofingiensis* to astaxanthin inducing conditions, secondary carotenoids increased considerably, yet the content of total carotenoids showed only a slight increase accompanied with a severe decrease of primary carotenoids [14, 18, 32, 41]. In this context, the increase of secondary carotenoids including astaxanthin in *C. zofingiensis* is not likely attributed to the enhancement of overall carotenoid flux, as suggested in *H. pluvialis*, but instead caused by rerouting the carotenoid flux from primary carotenoids to secondary carotenoids. This is also supported by the transcriptional regulation of carotenoid biosynthetic pathways in *C. zofingiensis* upon stress conditions: the MEP pathway and lycopene formation from IPP/DMAPP were not stimulated, while genes involved in the biosynthesis of astaxanthin and other secondary carotenoids are up-regulated and genes involved in lutein biosynthesis were down-regulated [18, 32, 39, 41]. It is worth noting that stress conditions that induce astaxanthin biosynthesis also trigger ROS buildup in *C. zofingiensis* [13, 244]. Moreover, addition of external ROS to *C. zofingiensis* cultures can promote accumulation of secondary carotenoids including astaxanthin [113, 244]. These also happen in *H. pluvialis* [274–276]. Therefore, it is believed that ROS are involved in the regulation of carotenogenesis for astaxanthin biosynthesis. Nevertheless, what ROS species are generated by these stress conditions and how algal cells sense ROS for triggering carotenogenesis still remain largely unknown.

*C. zofingiensis* synthesizes astaxanthin, yet at a level much lower than that in *H. pluvialis*, likely attributed to the differences between the two algae with respect to astaxanthin biosynthesis and regulation. First, during carotenogenesis, up-regulation of the MEP pathway and lycopene formation from IPP/DMAPP occurs in *H. pluvialis* but not in *C. zofingiensis* [41]. Therefore, the carbon flux to carotenoids is limited and cannot support high astaxanthin accumulation in *C. zofingiensis*. Second, unlike *H. pluvialis* BKT and CHYb that have strong activity in converting β-carotene in succession to astaxanthin without accumulating intermediates, *C. zofingiensis* BKT is able to convert zeaxanthin to astaxanthin yet not efficiently and CHYb has no activity in hydroxylating canthaxanthin to astaxanthin, leading to buildup of the intermediates canthaxanthin and adonixanthin [14, 36]. In this context, the astaxanthin biosynthetic pathway in *C. zofingiensis* performs less efficiently than that in *H. pluvialis*. Third, aside from acting on β-carotene and zeaxanthin, *C. zofingiensis* BKT is likely able to convert lutein to keto-lutein for accumulation [14, 36], further diverting carotenoid flux away from astaxanthin. Fourth, the synthesis of violaxanthin competes with astaxanthin formation for the substrate zeaxanthin in *C. zofingiensis* and thus may attenuate zeaxanthin availability for astaxanthin synthesis. Fifth, the astaxanthin esterase in *C. zofingiensis* is likely to accept more substrates for esterification than that in *H. pluvialis*, giving that *C. zofingiensis* accumulates esterified forms of astaxanthin, adonixanthin and keto-lutein [41, 107], while *H. pluvialis* produces only esterified astaxanthin [267, 277]. The non-specific substrate utilization of astaxanthin esterase may impair the availability of the enzyme for astaxanthin ester formation in *C. zofingiensis*. The less efficient esterification of astaxanthin likely in turn inhibits astaxanthin synthesis in a feedback manner.

## Crosstalk between TAG and astaxanthin biosynthesis in *C. zofingiensis*

Astaxanthin is a secondary metabolite that accumulates in *C. zofingiensis* and *H. pluvialis* under diverse stress conditions [13, 15, 16, 19, 32, 230]. These stress conditions also trigger synthesis of TAG, the major storage lipid, in the two algae. The concurrent synthesis of astaxanthin and TAG that share and may compete for the carbon precursor pyruvate, plus the presence of astaxanthin predominantly esterified with fatty acids, points to the potential crosstalk between TAG and astaxanthin biosynthesis in *C. zofingiensis* and *H. pluvialis*. It has been demonstrated that inhibition of de novo fatty acid synthesis by specific chemical inhibitors attenuated or even abolished astaxanthin accumulation in *H. pluvialis* [15, 16, 278]. Probably, the inhibition of fatty acid synthesis causes a shortage of fatty acids for astaxanthin esterification leading to attenuated accumulation of astaxanthin ester in *H. pluvialis* [278]. Furthermore, astaxanthin, once synthesized, is packed into the TAG-filled LDs for storage [109]. The inhibition of fatty acid synthesis causes a considerable reduction in TAG level and thus less LDs for accommodating astaxanthin, which likely in turn imposes feedback inhibition on astaxanthin synthesis and esterification. Therefore, it has been proposed that a certain level of TAG (or a certain number of LDs) is a prerequisite for astaxanthin synthesis and accumulation in *H. pluvialis* [15, 16, 278].

Intriguingly, the impaired astaxanthin accumulation caused by inhibition of de novo fatty acid synthesis that happens in *H. pluvialis* does not occur in *C. zofingiensis*; instead, astaxanthin showed an increase upon treatment of the inhibitor cerulenin [13, 14, 279]. The astaxanthin increase associated with cerulenin treatment is likely from transformation of other carotenoids rather than the shunt of carbon flux from fatty acids to carotenoid biosynthetic pathways, as the total carotenoids showed little change, whereas β-carotene and canthaxanthin decreased [14]. One possible explanation for the contrary responses of astaxanthin to cerulenin treatment in *C. zofingiensis* and *H. pluvialis* is that the former synthesizes considerably lower astaxanthin than the latter and, therefore, needs fewer fatty acids and TAG-filled LDs for astaxanthin esterification and storage, respectively. Consistent with this, *C. zofingiensis* has lower ratios of astaxanthin/total fatty acids (TFA) and astaxanthin/TAG than *H. pluvialis* [14]. Nevertheless, both ratios in *C. zofingiensis* showed drastic increases upon cerulenin treatment and their values exceeded that in *H. pluvialis*, suggesting astaxanthin biosynthesis and accumulation in *C. zofingiensis* may not be restricted by the availability of fatty acids or TAG [14]. Probably, cerulenin treatment induces ROS production [13], which in turn serves as a signal to stimulate astaxanthin biosynthesis in *C. zofingiensis*.

The inhibition of astaxanthin biosynthesis, on the other hand, has little effect on TAG accumulation in *C. zofingiensis* [14]. This resembles the observations in *H. pluvialis* [16] and points to the fact that TAG biosynthesis is independent of astaxanthin biosynthesis process in these two algae. It is reasonable as many algae that do not synthesize astaxanthin are also able to accumulate TAG [3].

## Metabolic engineering for potential improvements in TAG and astaxanthin accumulation by *C. zofingiensis*

Both TAG and astaxanthin are secondary metabolites and generally accumulate in *C. zofingiensis* under stress conditions rather than favorable growth conditions. These abiotic stress conditions, nevertheless, impair algal growth and thus the production of TAG and astaxanthin. Genetic engineering of *C. zofingiensis* has the potential to allow the alga to synthesize more target products and accumulate even under non-stress conditions. Many candidate genes with engineering potential for improving TAG and/or astaxanthin production have been identified, which can be achieved by such strategies as ‘pushing’, ‘pulling’ and ‘protection’ summarized in Table 6.

**Table 6:** List of potential engineering targets for enhancing the synthesis of TAG and/or astaxanthin in *C. zofingiensis*

Gene Name	Gene ID	Function description	Expression pattern upon ND	Strategies
*Metabolic engineering for TAG improvements*				
PDHC	E1A (Cz03g08090), E1B (Cz01g37230)	Produces acetyl-CoA from pyruvate	Up-regulated	Overexpression
ACS	ACS2 (Cz12g10100)	Produces acetyl-CoA from acetate	Up-regulated	Overexpression
FAT	Cz04g05080	Releases FFAs from acyl-ACPs	Up-regulated	Overexpression
PGD1	Cz01g38020	Releases FFAs from MGDG	Up-regulated	Overexpression
Putative membrane lipid lipases	Cz02g15090, Cz03g14190, Cz01g06170, Cz12g10010	Releases FFAs from membrane lipids	Up-regulated	Overexpression
LACS	LACS2 (Cz11g20120)	Converts FFAs to acyl-CoAs for TAG synthesis	Up-regulated	Overexpression
GPDH	GPDH2 (Cz04g17090)	Converts DHAP to G3P for TAG synthesis	Up-regulated	Overexpression
GK	GK2 (Cz18g13070)	Converts glycerol to G3P for TAG synthesis	Up-regulated	Overexpression
GPAT	GPAT2 (Cz09g31330)	Transfers the acyl moiety from acyl-CoAs to G3P for LPA synthesis	Up-regulated	Overexpression
LPAAT	LPAAT1 (Cz16g02090), LPAAT2 (Cz04g14150)	Transfers the acyl moiety from acyl-CoAs to LPA for PA synthesis	Up-regulated	Overexpression
DGAT	DGAT1A (Cz06g05010), DGTT5 (Cz09g08290)	Transfers the acyl moiety from acyl-CoAs to DAG for TAG synthesis	Up-regulated	Overexpression
MLDP	Cz04g29220	Stabilizes LDs for TAG storage	Up-regulated	Overexpression
Putative TAG lipases	SDP1 (Cz05g29160), Cz05g31060	Hydrolysis of TAG	Down-regulated	Suppression
LACS	LACS5 (Cz12g27140)	Converts FFAs to acyl-CoAs for β-oxidation	Little change	Suppression
*Metabolic engineering for astaxanthin improvements*				
DXS	Cz02g35280	Involved in the MEP pathway for IPP/DMAPP	Little change	Overexpression
DXR	Cz07g19130	Involved in the MEP pathway for IPP/DMAPP	Little change	Overexpression
HDR	Cz05g23010	Involved in the MEP pathway for IPP/DMAPP	Little change	Overexpression
PSY	Cz05g32220	Condenses GGPP to phytoene	Little change	Overexpression
PDS	Cz02g32280	Converts phytoene to phytofluene	Little change	Overexpression
LCYb	Cz12g10170	Synthesizes β-carotene from lycopene	Up-regulated	Overexpression
LCYe	Cz09g18310	Synthesizes α-carotene from lycopene	Down-regulated	Suppression
BKT	BKT1 (Cz13g13100)	Converts β-carotene and zeaxanthin to canthaxanthin and astaxanthin, respectively	Up-regulated	Overexpression
CHYb	Cz12g16080	Converts β-carotene to zeaxanthin	Up-regulated	Overexpression
*Metabolic engineering for both TAG and astaxanthin improvements*				
MYB	Cz10g24240, Cz06g23090	MYB transcriptional factor predicted to regulate both TAG and astaxanthin synthesis	Up-regulated	Overexpression
bHLH	Cz03g20070, UNPLg00160	bHLH transcriptional factor predicted to regulate both TAG and astaxanthin synthesis	Up-regulated	Overexpression
bZIP	Cz15g21170, UNPLg00449	bZIP transcriptional factor predicted to regulate both TAG and astaxanthin synthesis	Up-regulated	Overexpression
G6PD	Cz06g12080, Cz03g12030	Provides NADPH	Up-regulated	Overexpression
6PGD	Cz05g06160	Provides NADPH	Up-regulated	Overexpression
ME	Cz15g18140	Produces pyruvate and NADPH from malate	Up-regulated	Overexpression

### Metabolic engineering for TAG improvement

Acetyl-CoA is the precursor of de novo fatty acid synthesis and increasing acetyl-CoA supply has proven to be a feasible ‘pushing’ strategy for promoting fatty acid synthesis and TAG accumulation in several algae [280–282]. *C. zofingiensis* mainly employs the chloroplastic PDHC and ACS, which are transcriptionally up-regulated by ND, to produce acetyl-CoA for de novo fatty acid synthesis. Overexpression of them has the potential to enhance TAG synthesis in *C. zofingiensis*. The fatty acyls used in the Kennedy pathway for TAG assembly are in the form of acyl-CoAs; they can be converted from the de novo synthesized acyl-ACPs mediated by the combination of FAT and LACS or from turnover of membrane lipids mediated by the combination of membrane lipid lipase and LACS. In *C. zofingiensis*, *FAT*, *LACS2*, *PGD1* and certain other putative membrane lipid lipase genes (Cz02g15090, Cz03g14190, Cz01g06170 and Cz12g10010) are considerably up-regulated by TAG inducing conditions and may represent promising engineering targets. As a support, heterologous expression of *C. zofingiensis LACS2* in *N. oceanica* or yeast has proven to promote TAG synthesis [151]. Heterologous expression of Cz01g06170 or Cz12g10010 also promoted TAG synthesis in yeast [40]. G3P is the other precursor used for TAG assembly and its generation can be either from glycerol catalyzed by glycerol kinase (GK) or from dihydroxyacetone phosphate (DHAP) catalyzed by G3P dehydrogenase (GPDH) [283]. *C. zofingiensis GPDH2* and *GK2* correlate well with TAG accumulation at the transcriptional level and are candidate gene targets with engineering potential. It has been reported in *C. reinhardtii* that overexpression of *GPD2*, a homolog to *C. zofingiensis GPDH2*, promoted TAG accumulation substantially [284].

The acyltransferases GPAT, LPAAT and DGAT are appealing candidates of the ‘pulling’ strategy, as they provide strong pulling force to integrate fatty acids to the glycerol backbone for TAG assembly. It has been reported in several algae that overexpression of *GPAT* and/or *LPAAT* allowed synthesis of more TAG [181, 285–288]. In *C. zofingiensis*, *GPAT2*, *LPAAT1* and *LPAAT2* are stimulated to express under TAG inducing conditions and, therefore, are considered as promising gene targets. It has been demonstrated that engineering *C. zofingiensis* via overexpressing *GPAT2* led to enhanced TAG accumulation [37]. Compared to GPAT and LPAAT, DGAT is believed to catalyze the rate-limiting step in TAG synthesis and represents a particularly interesting target for manipulating not only TAG content but also the fatty acid composition of TAG. Substantial TAG improvements (up to over twofold increase) by overexpressing *DGAT* genes have been achieved in many algae including *C. reinhardtti* [188, 289], *N. oceanica* [189, 194–196], and *P. tricornutum* [99, 198, 199, 290, 291], *T. pseudonana* [292], *Scenedesmus obliquus* [293] and *Neochloris oleoabundans* [294]. Of the ten *C. zofingiensis DGAT* genes, *DGAT1A* and *DGTT5*, which are up-regulated considerably by ND, possess strong activities towards a broad range of substrates for TAG synthesis [189]. Overexpression of these two genes in *C. zofingiensis* may have the potential to boost TAG synthesis and production. Moreover, MLDP, the major structural protein of LDs that has been shown to promote TAG synthesis in yeast and *C. reinhardtii* via overexpression [40], is also a candidate target for TAG improvement in *C. zofingiensis*.

It is worth noting that TAG level in algae depends on not only biosynthesis but also catabolism. Protecting TAG against degradation represents another option to promote algal TAG level. Several TAG lipases from algae have been characterized and suppression of these lipase genes has proven to successfully enhance TAG content [207, 209, 210, 295, 296]. In *C. zofingiensis*, SDP1 and another putative TAG lipase encoded by Cz02g29090 are believed to participate in TAG degradation; suppression of them via knockdown or knockout should be beneficial to TAG accumulation. Moreover, the fatty acids released from TAG can enter peroxisomes and undergo degradation via the fatty acid β-oxidation process [159]. Inactivation of this process hinders TAG degradation and thus can increase TAG content, which has been achieved in *C. reinhardtii* by inactivating an *AOX* gene [160]. This should also be applicable to *C. zofingiensis* via suppressing *AOX* genes or *LACS5* that encodes a peroxisomal enzyme response for converting free fatty acids to acyl-CoAs ready for downstream oxidation.

### Metabolic engineering for astaxanthin improvement

In *C. zofingiensis*, the MEP pathway, not stimulated under astaxanthin inducing conditions, is likely a limiting factor for astaxanthin synthesis. Therefore, overexpression of *DXS*, *DXR*, and *HDR*, which are considered as key genes involved in controlling carbon flux of the MEP pathway [297–299], may provide sufficient precursors IPP/DMAPP and push them to carotenoids for synthesizing more astaxanthin. Manipulation of the pathway that converts IPP/DMAPP to lycopene via overexpressing involved genes (e.g., *PSY*, *PDS*, *ZDS*) may also have the potential to enhance astaxanthin synthesis, as it can pull IPP/DMAPP away from sterols and other isoprenoids to carotenoids. In support of this, heterologous expression of *C. zofingiensis PSY* gene in *C. reinhardtii* resulted in enhanced carotenoid synthesis [239]. In addition, overexpression of *PDS* in *C. zofingiensis* enabled the alga to produce more total carotenoids as well as astaxanthin [34].

LCYb and LCYe compete for lycopene and control the carotenoid flux between β-carotene and α-carotene derivatives and thus are promising engineering targets. Overexpression of *LCYb* may pull more carotenoid flux to β-carotene for downstream astaxanthin synthesis. On the other hand, suppressing *LCYe* can impair biosynthesis of lutein and its keto derivative keto-lutein, particularly the latter that accumulates under astaxnathin inducing conditions [19, 41], and thus has the potential to shunt the carotenoid flux from the α-carotene branching route to the β-carotene branching route. Considering the low efficiency of *C. zofingiensis* BKT in ketolating zeaxanthin to astaxanthin and failure of CHYb in hydroxylating canthaxanthin to astaxanthin [14], introduction of a BKT converting zeaxanthin to astaxanthin efficiently and a CHYb with strong activity in converting canthaxanthin to astaxanthin is essential for minimizing the buildup of intermediates and pulling carotenoid flux to the end product astaxanthin, and represents a promising engineering strategy to increase astaxanthin content as well as purity. The truncated *C. reinhardtii* BKT and *H. pluvialis* CHYb are such enzyme pair for maximizing astaxanthin production from β-carotene, which have been evidenced in vascular plants [253, 270]. Overexpressing a gene responsible for astaxanthin esterification also has the potential to boost astaxanthin production, because it can on the one hand sequester free astaxanthin thereby releasing the product feedback inhibition on astaxanthin biosynthesis and on the other hand protect astaxanthin against degradation since esterified astaxanthin is more stable than free astaxanthin. Of course, such a gene needs to be characterized first. Furthermore, the carotenoids β-carotene and zeaxanthin that are synthesized in the chloroplast have to be exported out for astaxanthin biosynthesis. Therefore, promoting transport of carotenoid precursors across the chloroplast membranes is beneficial to astaxanthin synthesis.

### Metabolic engineering for both TAG and astaxanthin improvements

The biosynthesis of TAG and astaxanthin each involves multiple coordinated steps. While manipulating a single gene can hardly obtain satisfactory increase of target products, multigene engineering is not easy to achieve in algae. Transcriptional factors (TFs) are a group of regulators that bind with certain upstream elements of their target genes and control their transcriptional expression. It has been reported in algae that TF manipulation is able to up-regulate multiple genes involved in lipid metabolism simultaneously and boost TAG accumulation [300–303]. In *C. zofingiensis*, there are several TFs of MYB (Cz10g24240 and Cz06g23090), bHLH (Cz03g20070 and UNPLg00160) and bZIP (Cz15g21170 and UNPLg00449) that are predicted to regulate both lipid metabolism for TAG synthesis and carotenogenesis for astaxanthin synthesis [37, 41]. Overexpression of these TF genes may have the potential to achieve TAG and astaxanthin improvements concurrently in *C. zofingiensis*. Overexpressing the genes involved in NADPH production is also a possible direction, as both biosynthetic pathways need NADPH as the reductant. Glucose-6-phosphate 1-dehydrogenase (G6PD), 6-phosphogluconate dehydrogenase (6PGD) and malic enzyme (ME) are such targets of engineering in *C. zofingiensis*. Moreover, blocking the carbon competing pathways, e.g., starch biosynthesis via suppression of ADP-glucose pyrophosphorylase, may reroute the carbon flux to lipids and carotenoids leading to enhanced accumulation of both TAG and astaxanthin.

## Conclusions

Despite substantial progresses achieved in the exploration of algal lipids for biodiesel production, the cost remains high and, therefore, restricts realization of commercial production of algae-derived biodiesel. Integrated production of algal lipids with value-added products represents one of the most promising strategies to improve the production economics of algal biodiesel. *C. zofingiensis*, able to grow robustly and achieve high biomass density under multiple trophic conditions, synthesizes TAG, the most energy-dense lipid ideal for making biodiesel, as well as astaxanthin, a high-value keto-carotenoid with broad applications. The simultaneous accumulation of TAG and astaxanthin allows integrated production of these two compounds by *C. zofingiensis* and thus has the potential to bring down the production cost of algal biodiesel. Since the release of chromosome-level whole genome sequence, many efforts have been made to better understand the pathways and regulation of TAG and astaxanthin biosynthesis in *C. zofingiensis*, which reveal distinctive features of this alga and help identify numerous gene targets for future engineering toward further improvements in TAG and/or astaxanthin levels. This has to rely on the establishment of more sophisticated genetic tools to allow easy and stable transformation, gene manipulation and genome editing of *C. zofingiensis*. Moreover, future directions also lie in development of next-generation culture systems for sustainable and cost-effective production of TAG-and-astaxanthin-rich biomass, and exploration of the state-of-art downstream processes for integrated production of TAG and astaxanthin. Breakthroughs occurring in these fields will greatly expand the production capacity and improve the production economics of *C. zofingiensis*.

## Data Availability

All data generated or analyzed during this study are included in this published article.

## Abbreviations

AACT: Acetoacetyl-CoA thiolase
AAT: Long-chain-alcohol *O*-fatty-acyltransferase
ACCase: Acetyl-CoA carboxylase
AdoMet: *S*-Adenosylmethionine
AOX: Acyl-CoA oxidase
BAT: Betaine lipid synthase
BKT: Beta-carotenoid ketolase
CDP-ME: 4-Diphosphocytidyl-2-C-methylerythritol
CDP-MEP: 4-Diphosphocytidyl-2-C-methyl-d-erythritol 2-phosphate
CDS: Phosphatidate cytidylyltransferase
CCT: Choline-phosphate cytidylyltransferase
CHK: Choline kinase
Cho: Choline
CHYb: Beta-carotenoid hydroxylase
CMK: 4-Diphosphocytidyl-2-C-methyl-d-erythritol kinase
CMS: 2-C-methyl-d-erythritol 4-phosphate cytidylyltransferase
CRTISO: Carotenoid isomerase
CYP97A: Cytochrome P450 beta hydroxylase
CYP97C: Cytochrome P450 epsilon hydroxylase
DAG: Diacylglycerol
DGAT: Diacylglycerol acyltransferase
DGD: Digalactosyldiacylglycerol synthase
DGDG: Digalactosyl diacylglycerol
DMAPP: Dimethylallyl pyrophosphate
DXR: 1-Deoxy-d-xylulose 5-phosphate reductoisomerase
DXP: 1-Deoxy-d-xylulose 5-phosphate
DXS: 1-Deoxy-d-xylulose 5-phosphate synthase
ECH: Enoyl-CoA hydratase
ECT: CDP-Ethanolamine synthase
ENR: Enoyl-ACP reductase
EPT/CPT: Ethanolaminephosphotransferase/cholinephosphotransferase
Eth: Ethanolamine
ETK: Ethanolamine kinase
GALE: UDP-galactose 4-epimerase
FAD: Fatty acid desaturase
FAT: Acyl-ACP thioesterase
FPP: Farnesyl diphosphate
FPPS: Farnesyl diphosphate synthase
GAP: Glyceraldehyde 3-phosphate
GGPP: Geranylgeranyl diphosphate
GGPPS: Geranylgeranyl diphosphate synthase
G3P: Glycerol-3-phosphate
GPAT: Glycerol-3-phosphate acyltransferase
GPP: Geranyl diphosphate
GPPS: Geranyl diphosphate synthase
HCS: Hydroxymethylglutaryl-CoA synthase
HAD: 3-Ketoacyl-ACP dehydratase
HCD: 3-Hydroxyacyl-CoA dehydrogenase
HDR: 4-Hydroxy-3-methylbut-2-en-1-yl diphosphate reductase
HDS: 4-Hydroxy-3-methylbut-2-en-1-yl diphosphate synthase
HGM-CoA: 3-Hydroxy-3-methylglutaryl-CoA
HMB-PP: (E)-4-Hydroxy-3-methyl-but-2-enyl pyrophosphate
IPP: Isopentenyl pyrophosphate
IPPI: Isopentenyl-diphosphate delta-isomerase
KAR: 3-Ketoacyl-ACP reductase
KAS: 3-Ketoacyl-ACP synthase
KATO: 3-Ketoacyl-CoA thiolase
LACS: Long-chain acyl-CoA synthetase
LCYb: Lycopene beta cyclase
LCYe: Lycopene epsilon cyclase
LD: Lipid droplet
LPA: Lysophosphatidic acid
LPAAT: Lysophosphatidic acid acyltransferase
MCS: 2-C-methyl-d-erythritol 2,4-cyclodiphosphate synthase
MCT: Malonyl-CoA:acyl carrier protein transacylase
Met: Methionine
MEcPP: 2-C-methyl-d-erythritol 2,4-cyclodiphosphate
MEP: 2-C-methylerythritol 4-phosphate
MIPS: Myo-inositol-1-phosphate synthase
MGD: Monogalactosyldiacylglycerol synthase
MGDG: Monogalactosyl diacylglycerol
MLDP: Major lipid droplet protein
NXS: Neoxanthin synthase
PA: Phosphatidic acid
PAP: Phosphatidate phosphatase
PC: Phosphatidylcholine
PDAT: Phospholipid:diacylglycerol acyltransferase
PDS: Phytoene desaturase
PE: Phosphatidylethanolamine
PEAMT: Phosphoethanolamine methyltransferase
PG: Phosphatidylglycerol
PGP: Phosphatidylglycerophosphatase
PGPS: Phosphatidylglycerophosphate synthase
PI: Phosphatidylinositol
PIS: Phosphatidylinositol synthase
PGD1: Plastid Galactoglycerolipid Degradation1
PSY: Phytoene synthase
SAD: Stearoyl-ACP desaturase
SAS: S-adenosylmethionine synthase
SQDG: Sulfoquinovosyl diacylglycerol
SDP1: Sugar-Dependent1 TAG lipase
TAG: Triacyglycerol
UGPase: UDP-glucose pyrophosphorylase
VDE: Violaxanthin de-epoxidase
ZDS: Zeta-carotene desaturase
ZEP: Zeaxanthin epoxidase
ZISO: Zeta-carotene isomerase
